# Frequency‐of‐seeing curves (psychometric functions) for perimetric stimuli in age‐related macular degeneration

**DOI:** 10.1111/opo.13396

**Published:** 2024-09-27

**Authors:** Jonathan Denniss, Helen C. Baggaley, Andrew T. Astle

**Affiliations:** ^1^ School of Optometry and Vision Science University of Bradford Bradford UK; ^2^ Optometry Unit, Department of Ophthalmology Nottingham University Hospitals NHS Trust Nottingham UK; ^3^ Independent Researcher Nottingham UK

**Keywords:** age‐related macular degeneration, frequency‐of‐seeing, microperimetry, perimetry, psychometric function

## Abstract

**Purpose:**

Frequency‐of‐seeing (FoS) curves (psychometric functions) for perimetric stimuli have been widely used in computer simulations of new visual field test procedures. FoS curves for age‐related macular degeneration (AMD) are not available in the literature and are needed for the development of improved microperimetry test procedures, which are of particular interest for use as clinical trial endpoints.

**Methods:**

Data were refitted from a previous study to generate FoS curves for 20 participants with AMD, each tested at nine locations within the central 10°. Stimulus parameters, background luminance and dB scale were matched to the MAIA‐2 microperimeter, and stimuli were presented in a method of constant stimuli to build up FoS curves over multiple runs. FoS curves were fitted with a modified cumulative Gaussian function. The relationship between sensitivity and slope of fitted FoS curves was modelled by robust linear regression, producing models both with and without an eccentricity parameter.

**Results:**

FoS curves were satisfactorily fitted to data from 174 visual field locations in 20 participants (age 65–83 years, 11 female). Each curve was made up of a median of 243 (range 177–297) stimulus presentations over a median of 12 (range 9–32) levels. Median sensitivity was 25.5 dB (range 3.8–31.4 dB). The median slope (SD of fitted function) was 1.6 dB (range 0.5–8.5 dB). As in previous studies of other conditions, the slope of fitted FoS curves increased as sensitivity decreased (*p* < 0.001).

**Conclusions:**

FoS are provided for participants with AMD, as well as models of the relationship between sensitivity and slope. These fitted models and data may be useful for computer simulation studies of microperimetry procedures. Full details of the fitted curves are provided as supporting information.

**Key points**

- Frequency‐of‐seeing curves are necessary for the development of new visual field test procedures by computer simulation.
- Frequency‐of‐seeing curves are provided for age‐related macular degeneration.
- Models of the relationships between sensitivity, slope and eccentricity are provided for frequency‐of‐seeing curves in age‐related macular degeneration.

## INTRODUCTION

With emerging candidate treatments for age‐related macular degeneration (AMD) there is increasing interest from researchers and regulatory bodies in visual function outcome measures for clinical trials. Microperimetry, also known as fundus‐controlled perimetry, is one way in which visual function can be measured in cases of AMD. Microperimetry is attractive as an endpoint metric for clinical trials as it promises to detect subtle changes, or the lack of, across a range of visual field locations which may be useful in monitoring the response to treatments aiming to halt, slow or reverse disease progression.^1^

Microperimetry is used to measure sensitivity across the central visual field with real‐time fundus tracking based on integral scanning laser ophthalmoscopy imaging. This enables microperimetry to measure sensitivity reliably even in patients who cannot hold their gaze steadily on a fixation target, and to map the position of a monocular preferred retinal locus.

Unlike conventional perimetry used in glaucoma and neurological conditions, current microperimetry for AMD uses a limited range of adaptive thresholding procedures which may not be optimised for emerging use cases. Due to the necessary trade‐off between time available to conduct a test and the precision, accuracy and number of threshold measurements made, all adaptive thresholding procedures are biased in some way. For example, measurements made using the 4–2 dB staircase procedure employed by some devices are biased towards the procedure's starting point. Alternatively, Bayesian procedures are biased by their prior probability density function. Different use cases may benefit from procedures biased in different ways. For example, clinical trials of potential new treatments for geographic atrophy may benefit from procedures that prioritise precise measurements in the low sensitivity range, whilst those aiming to use microperimetry for monitoring early stage AMD may prefer to prioritise measurement precision in the high sensitivity range. Therefore, it would be desirable to develop new microperimetry test procedures that are optimised for individual use cases, but to date, this has not been studied in‐depth.

In conventional perimetry, many test procedures have been developed through computer simulation prior to in‐human trials. This includes the Swedish Interactive Thresholding Algorithm (SITA) procedures perhaps most frequently encountered in clinical use^2^ and many others including, for example, suprathreshold approaches,^3^, ^4^ those aiming for faster or more precise threshold measurement^5^, ^6^, ^7^ or more specialised applications such as monitoring advanced loss,^8^ increasing spatial resolution^9^, ^10^ or integration with imaging information.^11^, ^12^ Computer simulation uniquely enables researchers to test candidate procedures efficiently, often optimising over large parameter spaces, and to measure accuracy and precision over many hundreds of repeated tests compared against known ‘ground truths’. Note that here the term *procedure* is used to mean the method by which the stimulus intensities to be presented, as well as the order in which they are presented, are chosen in order to measure sensitivity. This, therefore, does not include other possible changes to the test such as changes to the stimulus itself or testing apparatus.

Computer simulations of perimetric test procedures require a model of how the relevant human observers respond to stimuli of different intensities. In other words, we need to know the probability of a person with the condition of interest responding to stimuli of different intensities, given a particular underlying ‘true sensitivity’. These responses are modelled as psychometric functions, commonly known as frequency‐of‐seeing (FoS) curves in the perimetry literature.

FoS curves have been published for healthy eyes, glaucoma, ocular hypertension and optic neuritis.^13^, ^14^, ^15^, ^16^, ^17^ Henson et al.^16^ fitted models to their data to describe population average FoS curves for several conditions including glaucoma, ocular hypertension, optic neuritis and healthy eyes. These models have been widely used in simulations of perimetric test procedures and have been incorporated into the simulation functions of the Open Perimetry Interface.^18^

To the best of our knowledge, FoS curves for people with AMD have not been published, restricting the potential for improving microperimetry test procedures for this disease through computer simulation. Further, to the best of our knowledge, FoS curves have not been collected using the specific test parameters of a microperimetry device (background illumination, test area and stimulus parameters). We previously collected data underpinning FoS curves in people with AMD as part of a study on the relationship between microperimetric sensitivity and visual acuity.^19^ These data were collected under conditions mimicking a microperimeter. In that earlier study, we were interested only in the sensitivity and used the method of constant stimuli as a reliable way to measure sensitivity. The slopes of the functions were not reported or analysed. Here, the data from that study were refitted with an improved method and the relationship between the sensitivity and slope of the fitted functions was analysed. The full data on the fitted FoS curves are provided, along with fitted models relating sensitivity to slope for use in future simulations of microperimetry procedures.

## METHODS

The full details of the participants and data collection are given in the previous paper.^19^ Key details are replicated here for ease. The study was approved by the National Health Service National Research Ethics Service and adhered to the tenets of the Declaration of Helsinki. All participants gave written informed consent to take part.

### Participants

Twenty people (median age 74.5 years, range 65–83 years, 11 female, median visual acuity 0.31 logMAR, range 0.04–0.86 logMAR) with a confirmed clinical diagnosis of wet (*n* = 13) or dry (*n* = 7) AMD took part. Participants had no other known ocular pathology other than mild cataracts. All participants had stable foveal fixation according to a prior MAIA‐2 (CenterVue, icare‐world.com) microperimetry ‘Expert’ test. Beckman classification of AMD stage^20^ was ‘late’ in all 13 cases of wet AMD, ‘intermediate’ in six dry AMD cases and ‘early’ in one dry AMD case. Full details of each participant are given in Table 1 of Denniss et al.^19^

### Procedure

Briefly, after practice trials, each participant's visual field sensitivity was measured on a calibrated display (Display++, Cambridge Research Systems, crsltd.com/tools‐for‐vision‐science/calibrated‐displays/displaypp‐lcd‐monitor/) at nine spatial locations—(0°, 0°) and locations at 5° and 10° above, below, left and right of the fixation marker. The fixation marker was a broken cross, chosen to minimise masking effects on the central stimulus.^21^ Participants wore appropriate refractive correction for the screen distance (0.75–3 m to facilitate varied stimulus configurations used in the previous study but not relevant here). Head position was maintained by the use of a chin and forehead rest whilst steady central fixation was monitored by the experimenter via a mirror placed next to the screen to enable a clear view of the participant's eyes. The non‐tested eye was occluded with an opaque occluder.

Stimuli were 0.43° diameter circular luminance increments equivalent to the Goldmann III perimetric stimuli used in the MAIA‐2 microperimeter and other common devices. Other stimulus and background parameters were set to match those of the MAIA‐2 microperimeter, including 200 ms stimulus duration, dB scale for stimulus Weber contrast and 4 cd/m^2^ background luminance. FoS curves were measured using a method of constant stimuli built up over multiple 3–5 min runs with rest breaks in between. Participants responded to seen stimuli by pressing a button, with responses recorded within a 1750 ms window from stimulus offset. The next stimulus was presented 500–800 ms from the response or the end of the response window if there was no response. Testing was complete when satisfactory psychometric function fits were obtained with a minimum total of 175 presentations per test location spread across a minimum of nine contrast levels. Contrast levels were adjusted between runs by the experimenter in an attempt to span the range from 0% to 100% seen. Full details of the procedure are provided in the previous publication.^19^

### FoS curves

For this study, FoS curves were refitted to the percentage of stimuli seen at each contrast level for each location and participant separately. FoS curves were modelled as a modified cumulative Gaussian function as used in many recent simulation studies of perimetric procedures^6^, ^7^, ^8^, ^9^, ^10^, ^11^, ^12^:

(1) $$\Psi \left(x,t\right)=\mathrm{fp}+\left(1-\mathrm{fp}-\mathrm{fn})(1-G\left(x,t,s\right)\right)$$

In Equation (1), *fp* and *fn* represent the false‐positive and negative response rates defining the lower and upper asymptotes, respectively, and *G*(*x,t,s*) is the value at *x* of a cumulative Gaussian function with mean *t* and standard deviation *s*.

For this study, the FoS curves were refitted using a grid search maximum likelihood parameter estimation procedure, taking into account the number of presentations at each contrast level, such that levels with more presentations had a greater influence on the fit. The fitting procedure tried every possible combination of the mean (0.1 dB steps from 0.1 to 35.0 dB), standard deviation (0.1 dB steps from 0.1 to 12.0 dB) and false response rates (1% steps from 0% to 10%). The parameter combination with maximum likelihood was chosen. It was hypothesised that this method would improve the fits and allow the inclusion of more data, compared with the method used in the previous study which employed a fixed lower asymptote, freely varying upper asymptote and an optimisation procedure rather than the more computationally laborious grid search.^19^

As in the previous study, goodness‐of‐fit was assessed using a Monte Carlo method that generated empirical probabilities of the fitted function generating data with deviance as large or larger than the observed data. Higher probabilities therefore indicate a better fit. It was decided, prior to re‐fitting the FoS curves, that FoS curves with goodness‐of‐fit *p* < 0.05 would be excluded from further analysis, as were data for test locations at which the participant could not reliably (>75%) detect the highest intensity stimulus.

### Analysis

Individual FoS curve parameters, summary information and modelling of the relationship between sensitivity and slope in the FoS curves are reported. Because the slope of the FoS curve was determined by the standard deviation of the fitted cumulative Gaussian (*s* in Equation 1), slope was simply defined as the standard deviation of the fitted FoS curve in dB, such that larger dB slopes indicated flatter functions (more variable responses). Sensitivity was defined as the mean of the fitted FoS curve.

To estimate the population average relationship between sensitivity and slope of FoS curves, MM‐type robust linear regression^22^ was used as implemented in the lmrob() function of the *R* package robustbase.^23^ Robust regression was chosen as it minimises the influence of outliers that may be caused by poor FoS curve fits not captured by the exclusion criteria detailed above. A model containing only intercept and effect of sensitivity on slope was compared with a model with the added effect of eccentricity on slope by the Chi‐squared test of model quasi‐deviance.

Fitting of FoS curves and subsequent analyses were performed in *R* ^24^ version 4.2.1 (r‐project.org). Statistical significance was assumed at *p* < 0.05.

## RESULTS

FoS curves with satisfactory goodness‐of‐fit (*p* > 0.05) were obtained for a total of 176 of the 180 tested visual field locations across 20 participants with AMD. FoS curves from the remaining four tested visual field locations were excluded. Examination of the excluded data showed that in each case the participant was unable to reliably detect maximum luminance stimuli at that test location. A further two FoS curves were excluded for the same reason following manual inspection, despite goodness‐of‐fit *p*‐values > 0.05. Three of the excluded visual fields came from participant 10, with the remaining three being from participants 6, 11 and 18. This left a final sample of 174 FoS curves for further analysis. Plots of all FoS curves, including those excluded from further analysis are provided as Appendices S1 and S2, as are the fitted parameters for all curves.

The revised fitting procedure used here was able to fit 174 FoS curves satisfactorily; an improvement over the 164 using the previous method.^19^ Median goodness‐of‐fit was *p* = 0.81 (range 0.12–1.0); an improvement of 0.01 over the previous method (paired Wilcoxon signed‐rank test, *p* < 0.001).

The median total number of stimulus presentations per FoS curve was 243 (range 177–297), over a median of 12 (range 9–32) levels. Overall median sensitivity was 25.5 dB (range 3.8–31.4 dB), with individual participants' median sensitivity ranging from 19.6 to 28.8 dB. Overall median slope (standard deviation of fitted function) was 1.6 dB (range 0.5–8.5 dB), with individual participants' median slope ranging from 1.2 to 3.8 dB. The overall median false‐positive rate was 1% (range 0%–10%, though note this was capped at 10%), with individual participants' median false‐positive rates ranging from 0% to 10%. The overall median false‐negative rate was also 1% (range also 0%–10%, similarly capped at 10%), with individual participants' median false‐negative rates ranging from 0% to 9%.

**FIGURE 1:**
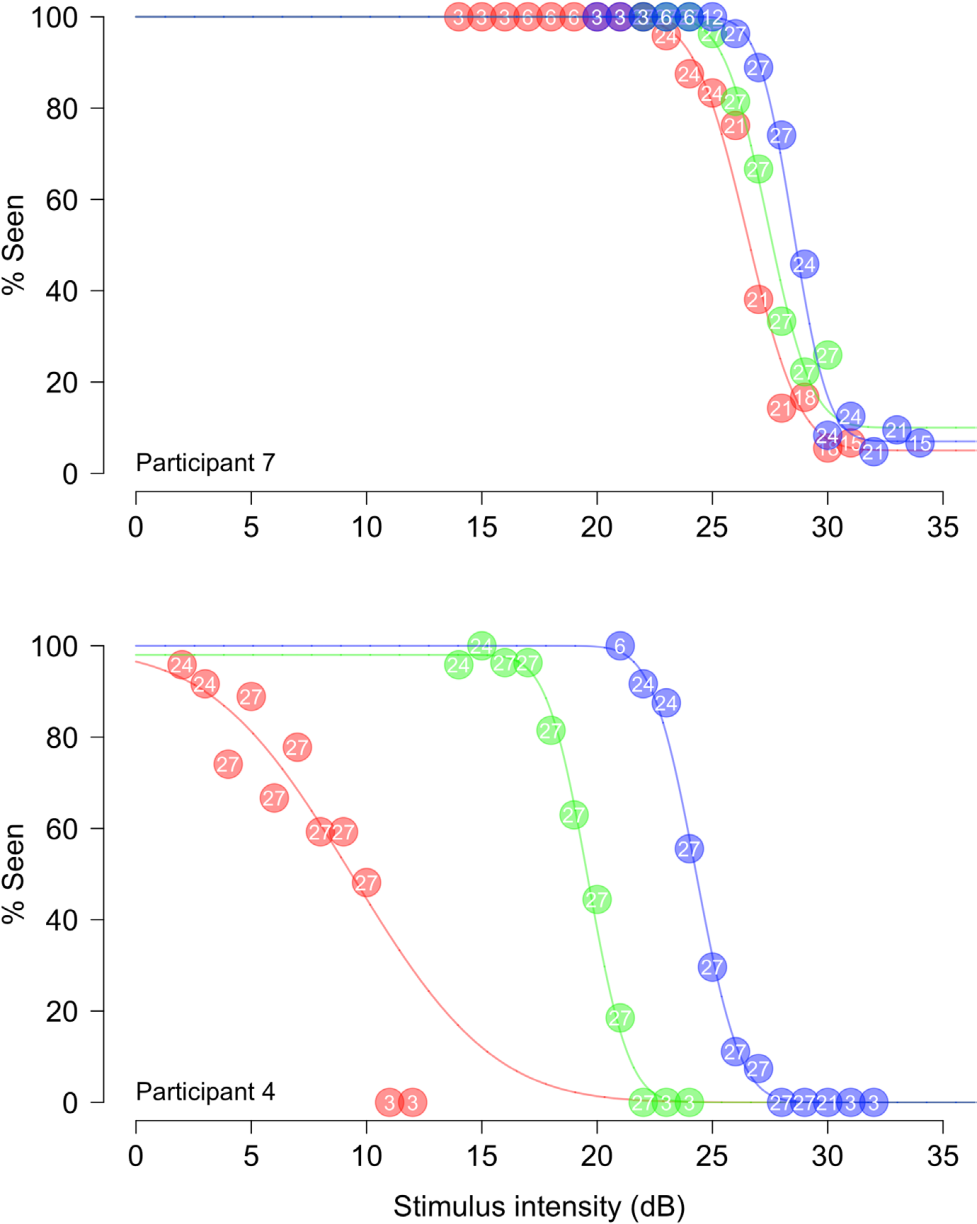
Example frequency‐of‐seeing (FoS) curves from the participant with the highest median sensitivity (participant 7, upper panel) and the participant with the lowest median sensitivity (participant 4, lower panel). Red, green and blue curves/points represent the lowest, median and highest sensitivity locations, respectively. Points show the percentage correct for stimuli at each contrast (intensity) level, with the number of presentations at each level given by the overlaid numbers on each point. Curves show the fitted FoS curves. These curves are plotted separately along with details of fitted parameters in Appendices S1 and S2.

Example FoS curves for the test locations with the highest, median and lowest sensitivity from the participants with the overall highest and lowest median sensitivity are shown in Figure 1.

**FIGURE 2:**
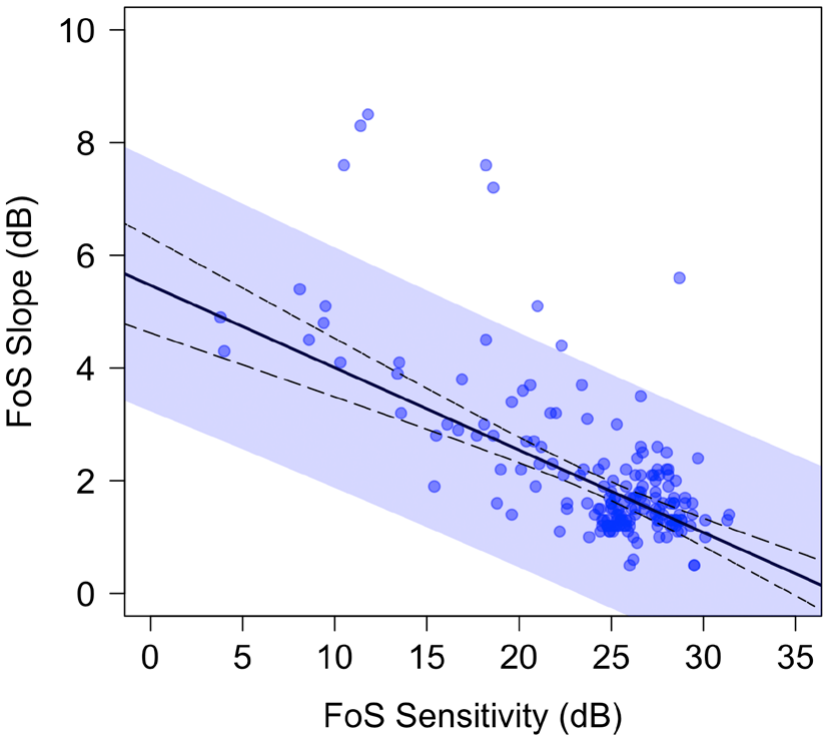
Sensitivity versus slope for the fitted frequency‐of‐seeing (FoS) curves. The solid black line shows the fit of Equation 2 to the data (95% confidence interval shown by black dashed lines). The blue‐shaded area shows the 95% prediction interval of the fit.

Figure 2 shows the relationship between sensitivity and slope across all tested locations and participants. The robust linear regression model shown in Figure 2 predicts the FoS curve slope from sensitivity only. For this approach the best‐fitting model was:

(2) $${\text{Slope}}_{i}=-0.15\;\left(\mathrm{SE}\;0.01\right)\times {\text{sensitivity}}_{i}+5.47\;\left(\mathrm{SE}\;0.28\right)+\varepsilon_{i}$$

where for participant *i*, ε_*i*_ represents random error, and slope, sensitivity and intercept are given in dB. Adjusted *R* ^2^ for this model was 0.60. Both sensitivity and intercept contributed significantly to the model (*p* < 0.001). The 95% prediction interval for this fit is shown by the blue‐shaded area in Figure 2 and had a mean full width of 4.27 dB (range 4.15 dB at 24.5 dB sensitivity to 4.47 dB at 0 dB sensitivity).

Incorporating the effects of eccentricity slightly improved the fit over the basic model including only threshold and effect of sensitivity (χ^2^(1) = 5.44, *p* = 0.02). For this approach, the best‐fitting model was:

(3) $$\begin{array}{c} {\text{Slope}}_{i}=-0.14\;\left(\mathrm{SE}\;0.01\right)\times {\text{sensitivity}}_{i}–0.03\;\left(\mathrm{SE}\;0.01\right)\\ \;\times \text{eccentricity}+5.54\;\left(\mathrm{SE}\;0.29\right)+\varepsilon_{i} \end{array}$$

where ε again represents random error for participant *i* and units are as for the first model other than eccentricity which is in degrees of visual angle. Adjusted *R* ^2^ for this model was 0.61. All parameters contributed significantly to the model (sensitivity and intercept *p* < 0.001, eccentricity *p* = 0.02).

## DISCUSSION

In this investigation, the data from our previous study were refitted using an improved method, resulting in successful FoS curve fits for 174 datasets from 20 participants with AMD tested at nine locations. Plots of the fits for all datasets and the associated fitted FoS curve parameters are provided as Appendices S1 and S2. Appendices S1 and S2 includes all datasets, including the six datasets that were excluded from further analysis.

The relationship between sensitivity and FoS curve slope in the data is shown in Figure 2. Also provided, in Equations 2 and 3, are models which can be used to generate FoS curves for a range of sensitivities in computer simulation studies. Equation 3 should be used in instances where it is desirable to take account of the small effect of eccentricity, but this has not typically been done in simulations of perimetric procedures to date. More commonly, simulations use models akin to Equation 2 that relate slope to sensitivity alone. Equation 2 is therefore plotted in Figure 2 and the 95% prediction interval for this fit is also provided.

The width of the 95% prediction interval shown in Figure 2 demonstrates the considerable between‐individual variation in the sensitivity–slope relationship. One possible source of this variation is differing response criteria between participants.^17^ If desired, simulation studies could sample from this prediction interval to simulate the between‐individual variation in FoS slopes for a given sensitivity. Again, this has not typically been done in simulation studies to date. It should be noted that most simulation studies are explorations of large parameter spaces, and therefore some simplification, such as the use of the population average FoS slope (Equation 2 in this study) is usually required for practical reasons.

Comparing the present data and model to those from previous studies of healthy participants, glaucoma, ocular hypertension and optic neuritis requires conversion between the different dB scales used. Rubinstein et al.^17^ used the Humphrey Field Analyser dB scale, whilst Henson et al.^16^ used the Henson CFA4000 perimeter, which has an equivalent dB scale to the Humphrey Field Analyser. In both of these studies, the conversion to their dB scale was MAIA dB‐4. After making this adjustment, the current FoS curves appear to be steeper and less dependent on sensitivity than those measured previously. These findings may be at least partially explained by the participants having to attend to a smaller visual field region (central 10°) than in these previous studies. Henson et al.^16^ presented stimuli at 12.7° for visually normal subjects or at varying locations within the 24–2 test pattern (extending 21°–27° from fixation) for participants with glaucoma or optic neuritis. Rubinstein et al. took the same approach as Henson et al.^16^ for participants with glaucoma.^17^ Henson et al.^16^ chose to log their slope values to achieve a linear fit. We saw no need to do this in the present data, nor in that of Rubinstein et al.^17^ Therefore, linear models were fitted directly to the data (which are themselves log–log scaled due to the dB scaling). The Henson et al.^16^ model produced very high slopes for low sensitivities that do not appear to be borne out in large‐scale clinical visual field data,^25^ leading most recent simulation studies using the Henson model to cap the slopes at 6 dB.^6^, ^7^, ^8^, ^9^, ^10^, ^11^, ^12^ A limitation of comparison to these previous studies is that the effect of the different background luminances cannot be fully equated as differences in adaptation state may affect the FoS curves. Further study is warranted to compare FoS curves directly from people with different conditions using precisely matched methodology. The current model and data may be more appropriate than those of the previous studies for simulations of microperimetry devices which tested a smaller region of visual field than conventional perimeters.

The choice of robust linear regression for modelling the relationship between sensitivity and slope is worthy of some discussion. It was decided prior to fitting the FoS curves to exclude data from locations where the goodness‐of‐fit achieved was *p* < 0.05, that is, datasets with a deviation larger than would be generated by the final fitted function in at least 1 of 20 repeats of the experiment. It was also decided to exclude datasets where there was evidence that the subject could not reliably (>75%) detect the highest intensity stimulus, because the goodness‐of‐fit could still be high when the FoS curve was essentially a flat line around 0% seen (e.g., Participant 10 location (0°, 10°) in Appendices S1 and S2). This method was successful in excluding the majority of poor fits as judged subjectively, but not quite all. The use of robust regression, therefore, minimised the influence of any remaining poor fits that resulted in outliers to the sensitivity–slope relationship, on the fitted models. This can be confirmed by looking subjectively at the fits for the six datasets that lie well above the prediction interval in Figure 2. All of these show subjectively poorer fits than the majority of the data, but they do not meet the pre‐determined conditions for exclusion. Nevertheless, the robust regression was able to minimise the effects of these outliers on the fitted models; thus producing models that represent the data from locations with good fits.

One limitation of the modelling is that repeated measures were not accounted for. Since data were included from up to nine locations per participant, there will be within‐participant effects unaccounted for by the models. We chose not to use a mixed modelling approach that would account for these effects because in the application of the model (computer simulations), the individual (per participant) slopes and intercepts generated by such an approach would be unknown, meaning that only the population average could be simulated. Should such an approach become useful in the future, these data can readily be reanalysed using the values supplied in Appendices S1 and S2. A further limitation of the data is that all of the participants fixated foveally; therefore, the effect on FoS curves of decentring the stimulus grid, for example, to centre on a preferred retinal locus when the fovea has been damaged, is not known. Many people with AMD do not have a stable central fixation, hence the requirement for fundus‐controlled perimetry. In case the participants also had unobserved, slightly unstable fixation that could have been controlled by a microperimeter, the effect of fixation control would have been to steepen slightly the observed FoS curves. The clinical characteristics of the study population are given in the table within the previous publication.^19^ The study population did not cover the full diversity of AMD, therefore the data may not be representative of all patients with AMD.^19^ Finally, as with previous studies of FoS curves in other conditions,^16^, ^17^ data for low sensitivities are more limited, meaning that the models are less certain for low sensitivities. Nevertheless, to the best of our knowledge, no other data on FoS curves for microperimetry stimuli are available for AMD patients, thus hindering the development of new microperimetry test procedures by computer simulation.

In conclusion, data and FoS curve fits for participants with AMD are provided under conditions similar to MAIA‐2 microperimetry. As in other conditions and in healthy eyes, FoS curves flatten as sensitivity reduces, meaning that sensitivity measurements can be expected to become more variable as sensitivity decreases. These FoS curves are generally steeper and flatten slightly less with sensitivity than those previously measured in individuals with glaucoma, though this may be related to the smaller, more central region of the visual field from which they were measured. Fitted parameters and plotted curves for all participants are provided as Appendices S1 and S2. These, along with the fitted models provided, may be useful for future computer simulation studies of microperimetry.

## AUTHOR CONTRIBUTIONS

**Jonathan Denniss:** Conceptualization (lead); data curation (lead); formal analysis (lead); investigation (lead); methodology (lead); validation (lead); visualization (lead); writing – original draft (lead); writing – review and editing (lead). **Helen C. Baggaley:** Data curation (supporting); investigation (supporting); writing – review and editing (supporting). **Andrew T. Astle:** Conceptualization (supporting); data curation (supporting); investigation (supporting); methodology (supporting); writing – review and editing (supporting).

## FUNDING INFORMATION

College of Optometrists Postdoctoral Fellowship (JD & ATA), National Institute for Health Research (NIHR) Postdoctoral Fellowship (ATA). This report presents independent research funded by the NIHR. The views expressed are those of the authors and not necessarily those of the NHS, the NIHR or the Department of Health.

## CONFLICT OF INTEREST STATEMENT

The authors declare no conflicts of interest.

## Supporting information


Appendix S1:

Appendix S2:
