# Supplementary material for: Frequency‐of‐seeing curves (psychometric functions) for perimetric stimuli in age‐related macular degeneration
Source: Ophthalmic Physiol Opt. 2024 Sep 27;45(1):301–7. doi: 10.1111/opo.13396 (PMC11629837; doi:10.1111/opo.13396)
Supplement: Supplementary file 2 — Appendix S2: [file OPO-45-301-s001.pdf]

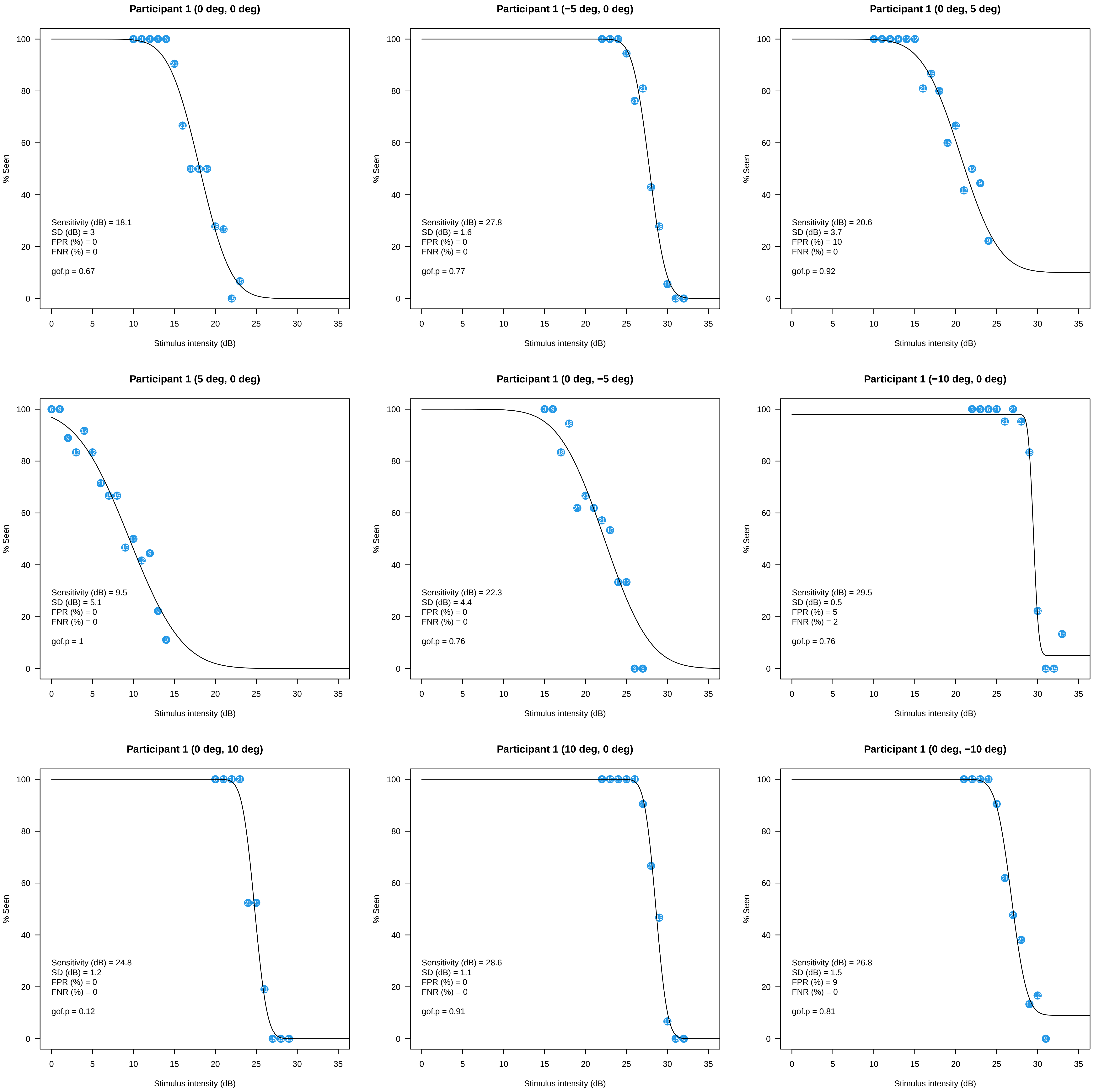
Participant 1 (0 deg, 0 deg)
Sensitivity (dB) = 18.1
SD (dB) = 3
FPR (%) = 0
FNR (%) = 0
gof.p = 0.67
Participant 1 (−5 deg, 0 deg)
Sensitivity (dB) = 27.8
SD (dB) = 1.6
FPR (%) = 0
FNR (%) = 0
gof.p = 0.77
Participant 1 (0 deg, 5 deg)
Sensitivity (dB) = 20.6
SD (dB) = 3.7
FPR (%) = 10
FNR (%) = 0
gof.p = 0.92
Participant 1 (5 deg, 0 deg)
Sensitivity (dB) = 9.5
SD (dB) = 5.1
FPR (%) = 0
FNR (%) = 0
gof.p = 1
Participant 1 (0 deg, −5 deg)
Sensitivity (dB) = 22.3
SD (dB) = 4.4
FPR (%) = 0
FNR (%) = 0
gof.p = 0.76
Participant 1 (−10 deg, 0 deg)
Sensitivity (dB) = 29.5
SD (dB) = 0.5
FPR (%) = 5
FNR (%) = 2
gof.p = 0.76
Participant 1 (0 deg, 10 deg)
Sensitivity (dB) = 24.8
SD (dB) = 1.2
FPR (%) = 0
FNR (%) = 0
gof.p = 0.12
Participant 1 (10 deg, 0 deg)
Sensitivity (dB) = 28.6
SD (dB) = 1.1
FPR (%) = 0
FNR (%) = 0
gof.p = 0.91
Participant 1 (0 deg, −10 deg)
Sensitivity (dB) = 26.8
SD (dB) = 1.5
FPR (%) = 9
FNR (%) = 0
gof.p = 0.81
% Seen
Stimulus intensity (dB)

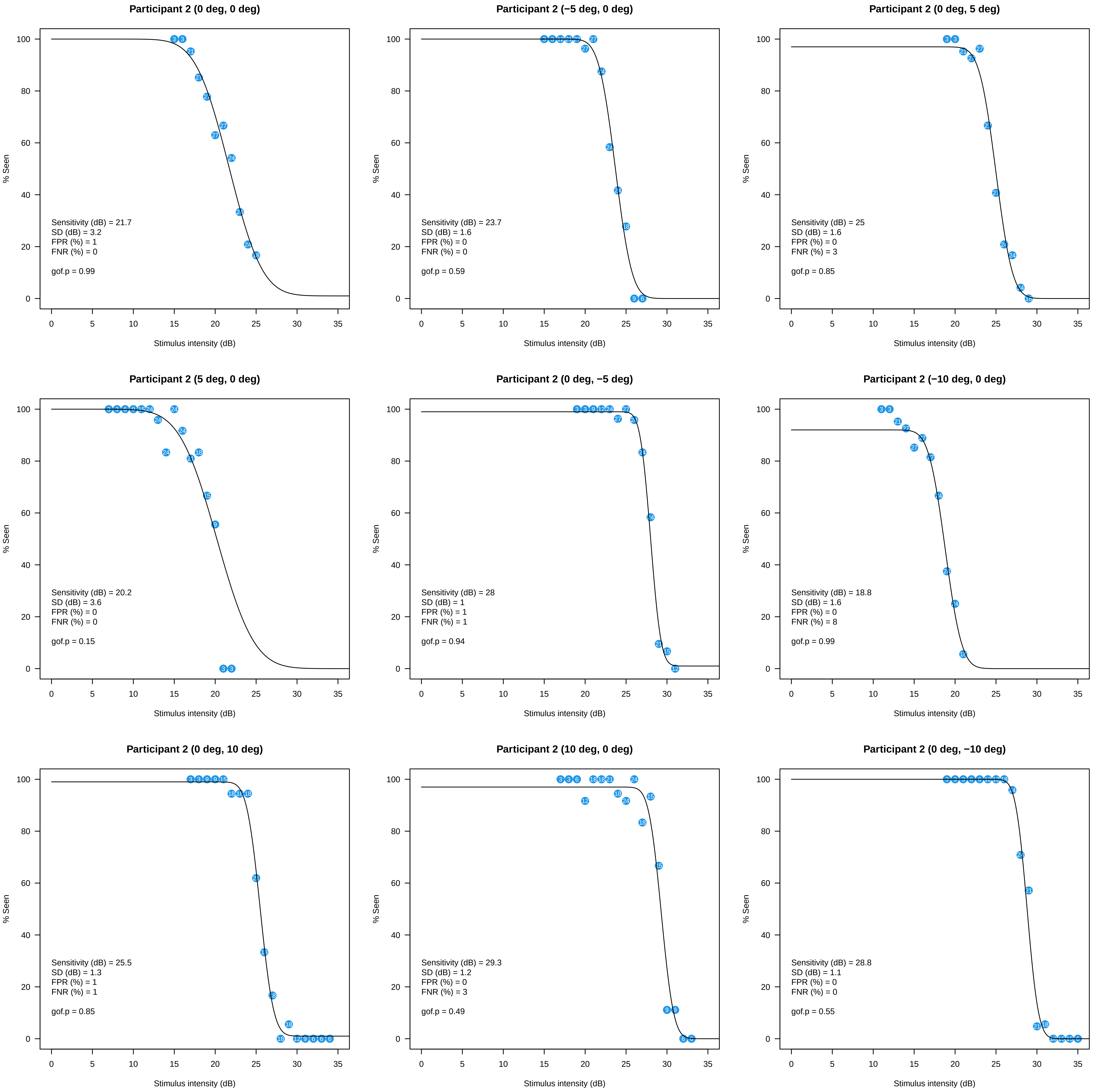

Participant 2 (0 deg, 0 deg)
Sensitivity (dB) = 21.7
SD (dB) = 3.2
FPR (%) = 1
FNR (%) = 0
gof.p = 0.99
Participant 2 (−5 deg, 0 deg)
Sensitivity (dB) = 23.7
SD (dB) = 1.6
FPR (%) = 0
FNR (%) = 0
gof.p = 0.59
Participant 2 (0 deg, 5 deg)
Sensitivity (dB) = 25
SD (dB) = 1.6
FPR (%) = 0
FNR (%) = 3
gof.p = 0.85
Participant 2 (5 deg, 0 deg)
Sensitivity (dB) = 20.2
SD (dB) = 3.6
FPR (%) = 0
FNR (%) = 0
gof.p = 0.15
Participant 2 (0 deg, −5 deg)
Sensitivity (dB) = 28
SD (dB) = 1
FPR (%) = 1
FNR (%) = 1
gof.p = 0.94
Participant 2 (−10 deg, 0 deg)
Sensitivity (dB) = 18.8
SD (dB) = 1.6
FPR (%) = 0
FNR (%) = 8
gof.p = 0.99
Participant 2 (0 deg, 10 deg)
Sensitivity (dB) = 25.5
SD (dB) = 1.3
FPR (%) = 1
FNR (%) = 1
gof.p = 0.85
Participant 2 (10 deg, 0 deg)
Sensitivity (dB) = 29.3
SD (dB) = 1.2
FPR (%) = 0
FNR (%) = 3
gof.p = 0.49
Participant 2 (0 deg, −10 deg)
Sensitivity (dB) = 28.8
SD (dB) = 1.1
FPR (%) = 0
FNR (%) = 0
gof.p = 0.55
% Seen
Stimulus intensity (dB)

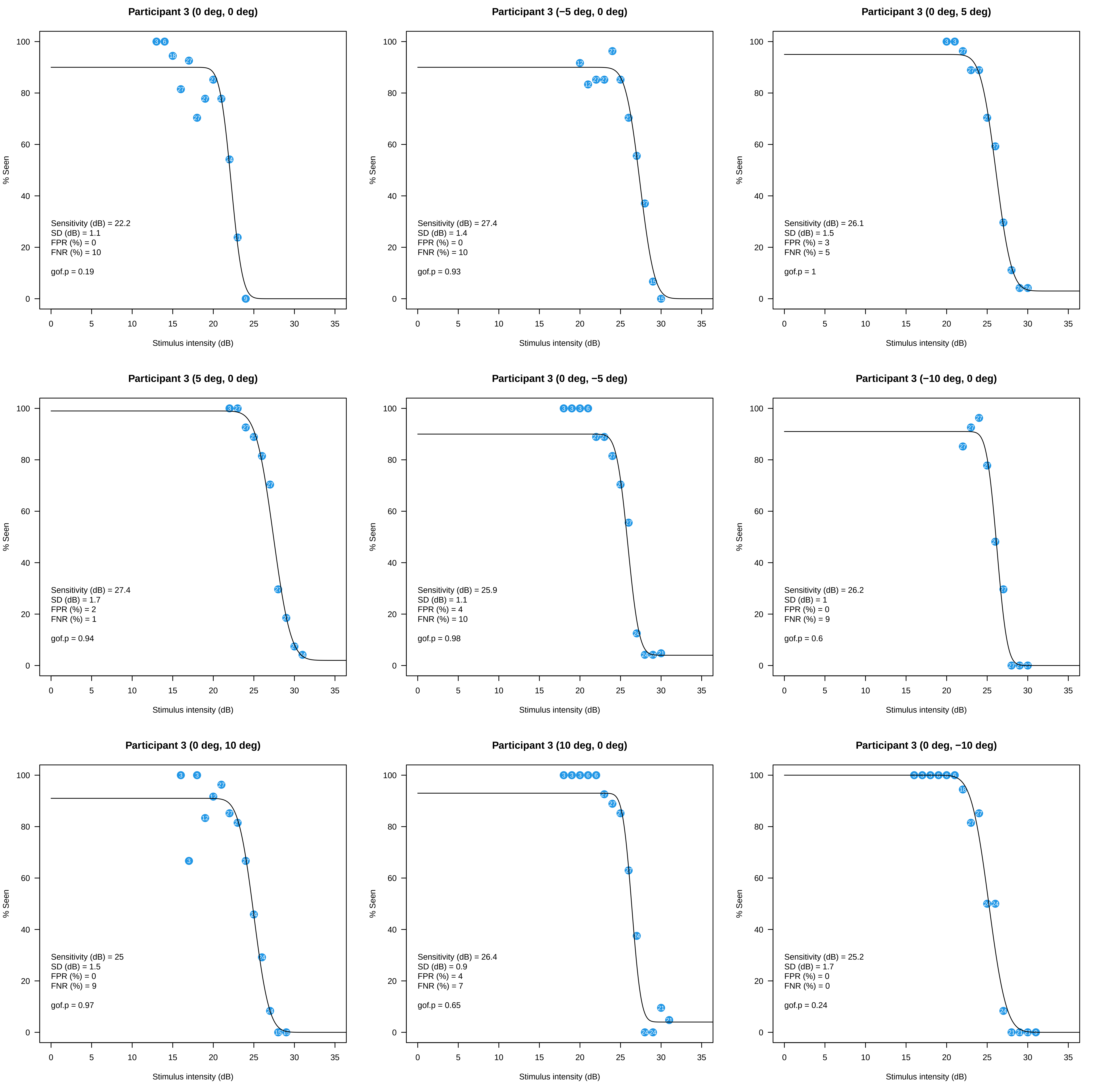

Participant 3 (0 deg, 0 deg)
Sensitivity (dB) = 22.2
SD (dB) = 1.1
FPR (%) = 0
FNR (%) = 10
gof.p = 0.19
Participant 3 (−5 deg, 0 deg)
Sensitivity (dB) = 27.4
SD (dB) = 1.4
FPR (%) = 0
FNR (%) = 10
gof.p = 0.93
Participant 3 (0 deg, 5 deg)
Sensitivity (dB) = 26.1
SD (dB) = 1.5
FPR (%) = 3
FNR (%) = 5
gof.p = 1
Participant 3 (5 deg, 0 deg)
Sensitivity (dB) = 27.4
SD (dB) = 1.7
FPR (%) = 2
FNR (%) = 1
gof.p = 0.94
Participant 3 (0 deg, −5 deg)
Sensitivity (dB) = 25.9
SD (dB) = 1.1
FPR (%) = 4
FNR (%) = 10
gof.p = 0.98
Participant 3 (−10 deg, 0 deg)
Sensitivity (dB) = 26.2
SD (dB) = 1
FPR (%) = 0
FNR (%) = 9
gof.p = 0.6
Participant 3 (0 deg, 10 deg)
Sensitivity (dB) = 25
SD (dB) = 1.5
FPR (%) = 0
FNR (%) = 9
gof.p = 0.97
Participant 3 (10 deg, 0 deg)
Sensitivity (dB) = 26.4
SD (dB) = 0.9
FPR (%) = 4
FNR (%) = 7
gof.p = 0.65
Participant 3 (0 deg, −10 deg)
Sensitivity (dB) = 25.2
SD (dB) = 1.7
FPR (%) = 0
FNR (%) = 0
gof.p = 0.24
% Seen
Stimulus intensity (dB)

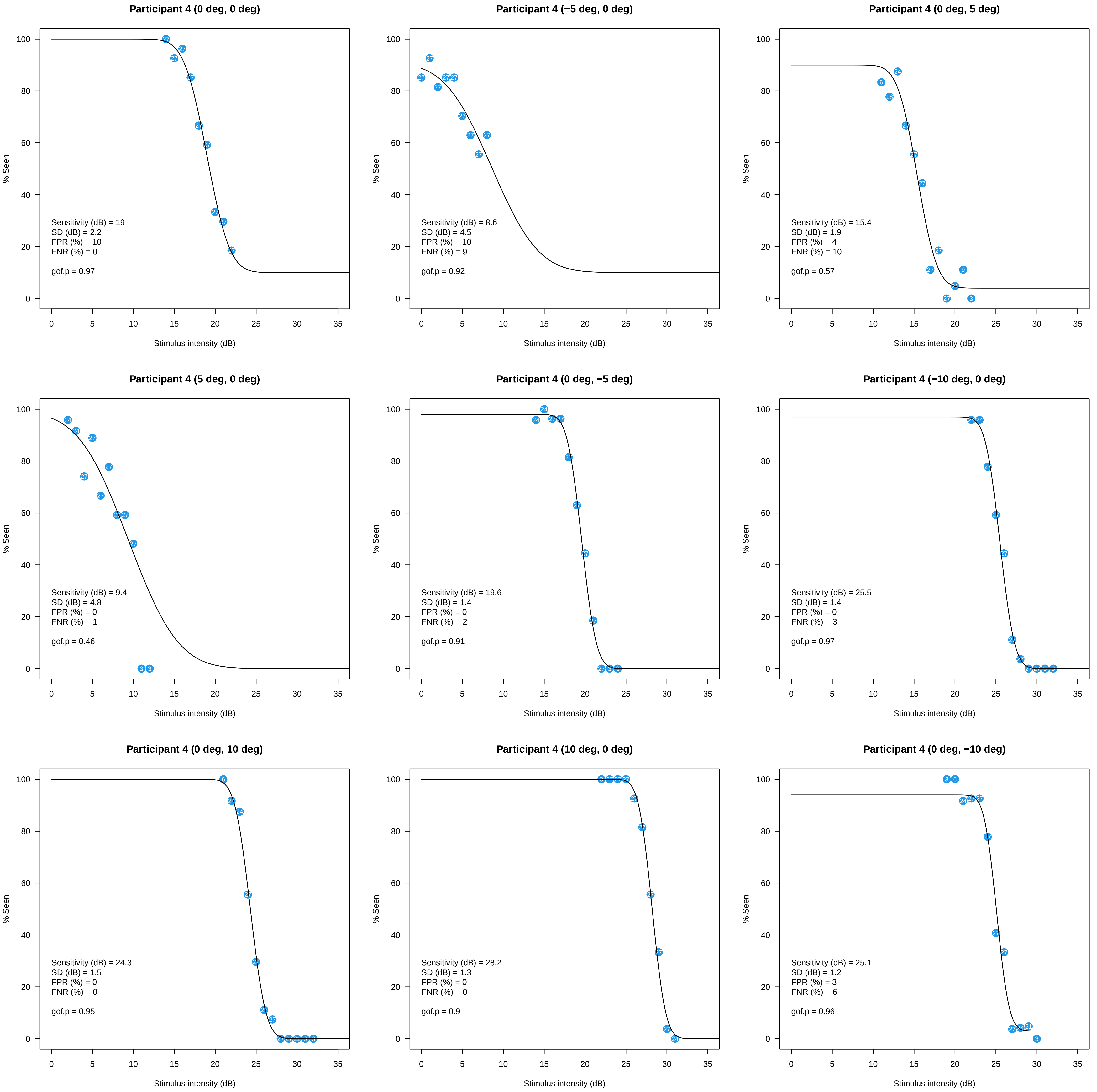
Participant 4 (0 deg, 0 deg)
Sensitivity (dB) = 19
SD (dB) = 2.2
FPR (%) = 10
FNR (%) = 0
gof.p = 0.97
Participant 4 (−5 deg, 0 deg)
Sensitivity (dB) = 8.6
SD (dB) = 4.5
FPR (%) = 10
FNR (%) = 9
gof.p = 0.92
Participant 4 (0 deg, 5 deg)
Sensitivity (dB) = 15.4
SD (dB) = 1.9
FPR (%) = 4
FNR (%) = 10
gof.p = 0.57
Participant 4 (5 deg, 0 deg)
Sensitivity (dB) = 9.4
SD (dB) = 4.8
FPR (%) = 0
FNR (%) = 1
gof.p = 0.46
Participant 4 (0 deg, −5 deg)
Sensitivity (dB) = 19.6
SD (dB) = 1.4
FPR (%) = 0
FNR (%) = 2
gof.p = 0.91
Participant 4 (−10 deg, 0 deg)
Sensitivity (dB) = 25.5
SD (dB) = 1.4
FPR (%) = 0
FNR (%) = 3
gof.p = 0.97
Participant 4 (0 deg, 10 deg)
Sensitivity (dB) = 24.3
SD (dB) = 1.5
FPR (%) = 0
FNR (%) = 0
gof.p = 0.95
Participant 4 (10 deg, 0 deg)
Sensitivity (dB) = 28.2
SD (dB) = 1.3
FPR (%) = 0
FNR (%) = 0
gof.p = 0.9
Participant 4 (0 deg, −10 deg)
Sensitivity (dB) = 25.1
SD (dB) = 1.2
FPR (%) = 3
FNR (%) = 6
gof.p = 0.96
% Seen
Stimulus intensity (dB)

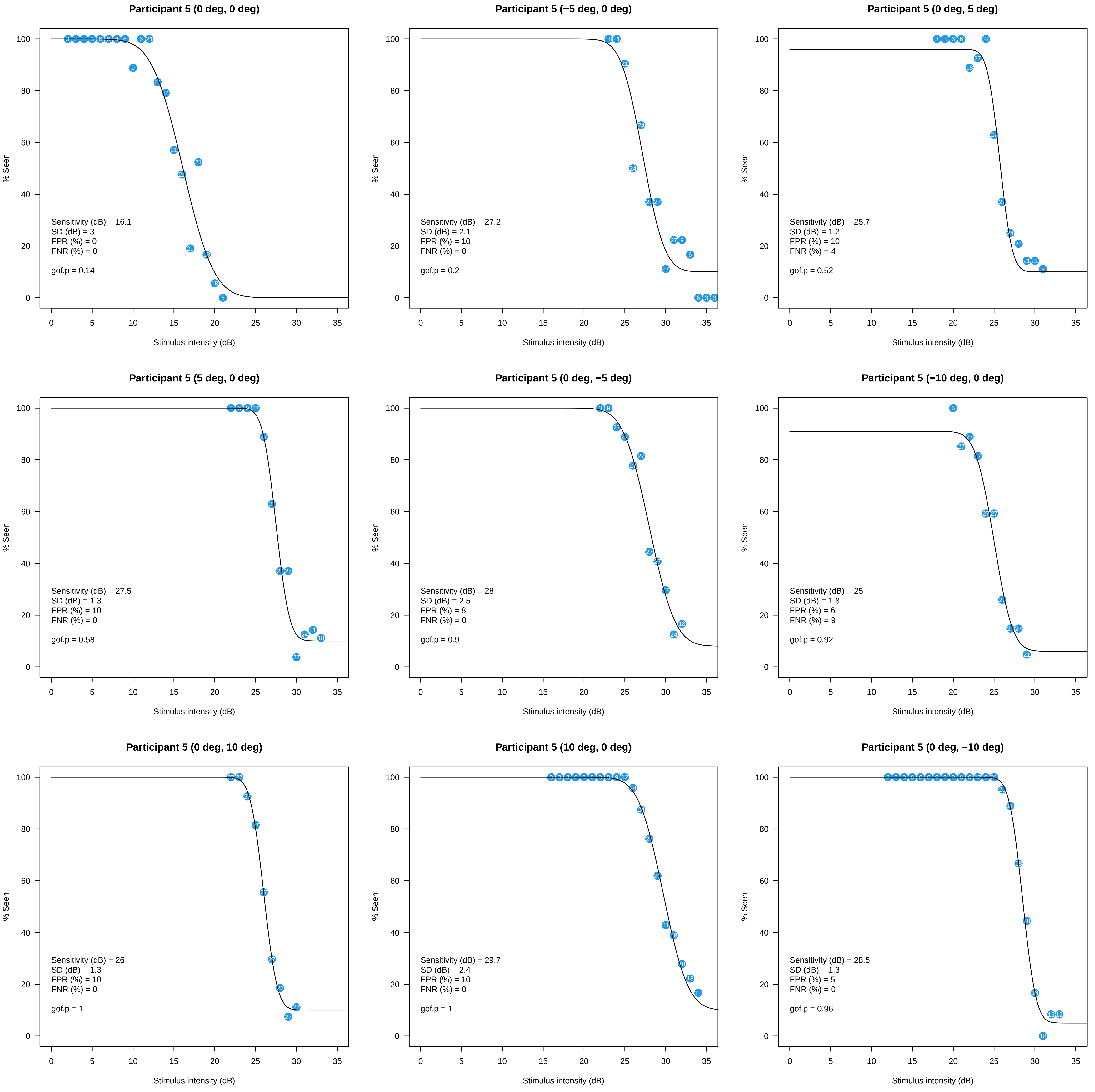

Participant 5 (0 deg, 0 deg)
Sensitivity (dB) = 16.1
SD (dB) = 3
FPR (%) = 0
FNR (%) = 0
gof.p = 0.14
Participant 5 (−5 deg, 0 deg)
Sensitivity (dB) = 27.2
SD (dB) = 2.1
FPR (%) = 10
FNR (%) = 0
gof.p = 0.2
Participant 5 (0 deg, 5 deg)
Sensitivity (dB) = 25.7
SD (dB) = 1.2
FPR (%) = 10
FNR (%) = 4
gof.p = 0.52
Participant 5 (5 deg, 0 deg)
Sensitivity (dB) = 27.5
SD (dB) = 1.3
FPR (%) = 10
FNR (%) = 0
gof.p = 0.58
Participant 5 (0 deg, −5 deg)
Sensitivity (dB) = 28
SD (dB) = 2.5
FPR (%) = 8
FNR (%) = 0
gof.p = 0.9
Participant 5 (−10 deg, 0 deg)
Sensitivity (dB) = 25
SD (dB) = 1.8
FPR (%) = 6
FNR (%) = 9
gof.p = 0.92
Participant 5 (0 deg, 10 deg)
Sensitivity (dB) = 26
SD (dB) = 1.3
FPR (%) = 10
FNR (%) = 0
gof.p = 1
Participant 5 (10 deg, 0 deg)
Sensitivity (dB) = 29.7
SD (dB) = 2.4
FPR (%) = 10
FNR (%) = 0
gof.p = 1
Participant 5 (0 deg, −10 deg)
Sensitivity (dB) = 28.5
SD (dB) = 1.3
FPR (%) = 5
FNR (%) = 0
gof.p = 0.96
% Seen
Stimulus intensity (dB)

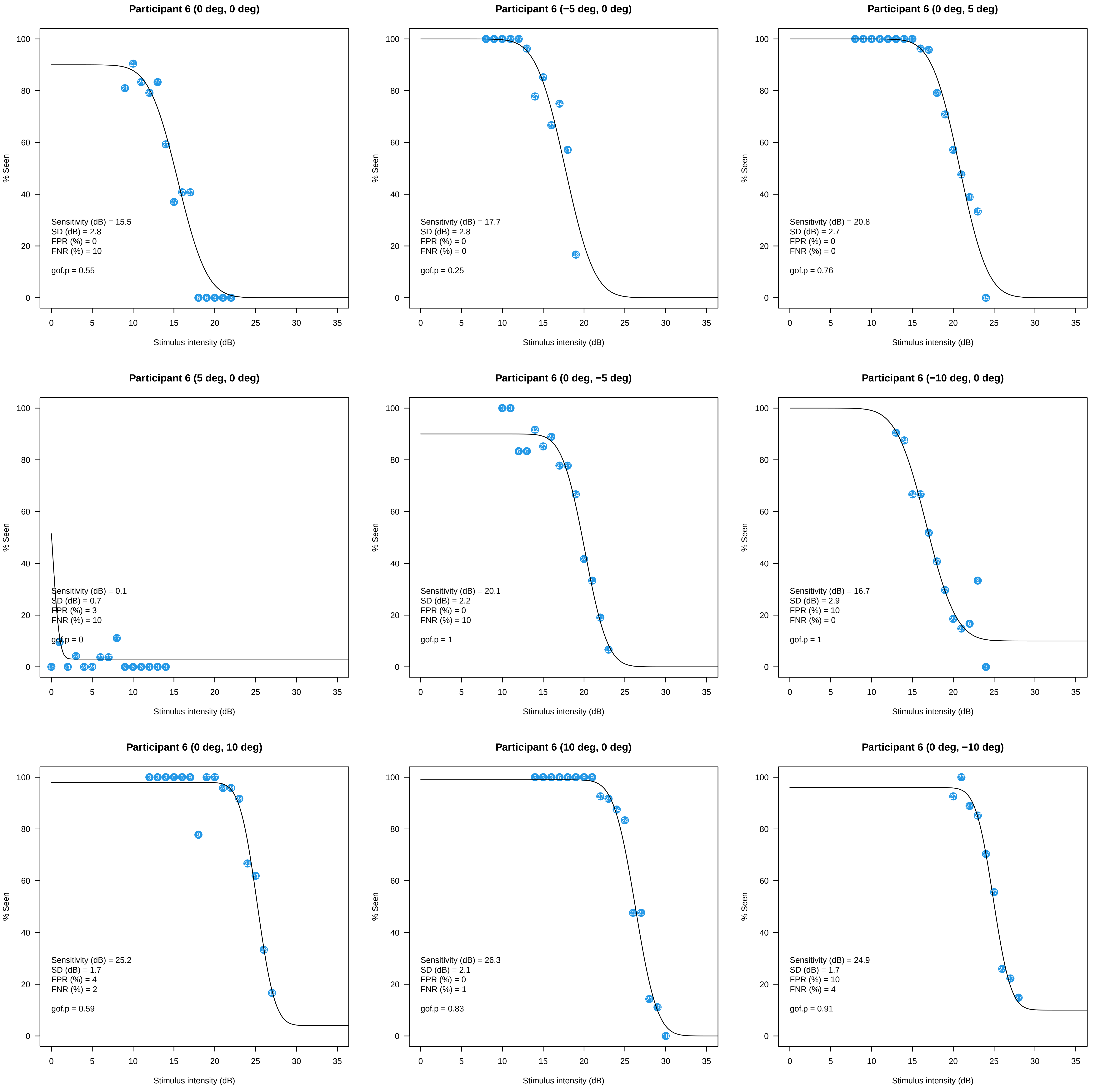
Participant 6 (0 deg, 0 deg)
Sensitivity (dB) = 15.5
SD (dB) = 2.8
FPR (%) = 0
FNR (%) = 10
gof.p = 0.55
Participant 6 (−5 deg, 0 deg)
Sensitivity (dB) = 17.7
SD (dB) = 2.8
FPR (%) = 0
FNR (%) = 0
gof.p = 0.25
Participant 6 (0 deg, 5 deg)
Sensitivity (dB) = 20.8
SD (dB) = 2.7
FPR (%) = 0
FNR (%) = 0
gof.p = 0.76
Participant 6 (5 deg, 0 deg)
Sensitivity (dB) = 0.1
SD (dB) = 0.7
FPR (%) = 3
FNR (%) = 10
gof.p = 0
Participant 6 (0 deg, −5 deg)
Sensitivity (dB) = 20.1
SD (dB) = 2.2
FPR (%) = 0
FNR (%) = 10
gof.p = 1
Participant 6 (−10 deg, 0 deg)
Sensitivity (dB) = 16.7
SD (dB) = 2.9
FPR (%) = 10
FNR (%) = 0
gof.p = 1
Participant 6 (0 deg, 10 deg)
Sensitivity (dB) = 25.2
SD (dB) = 1.7
FPR (%) = 4
FNR (%) = 2
gof.p = 0.59
Participant 6 (10 deg, 0 deg)
Sensitivity (dB) = 26.3
SD (dB) = 2.1
FPR (%) = 0
FNR (%) = 1
gof.p = 0.83
Participant 6 (0 deg, −10 deg)
Sensitivity (dB) = 24.9
SD (dB) = 1.7
FPR (%) = 10
FNR (%) = 4
gof.p = 0.91
% Seen
Stimulus intensity (dB)

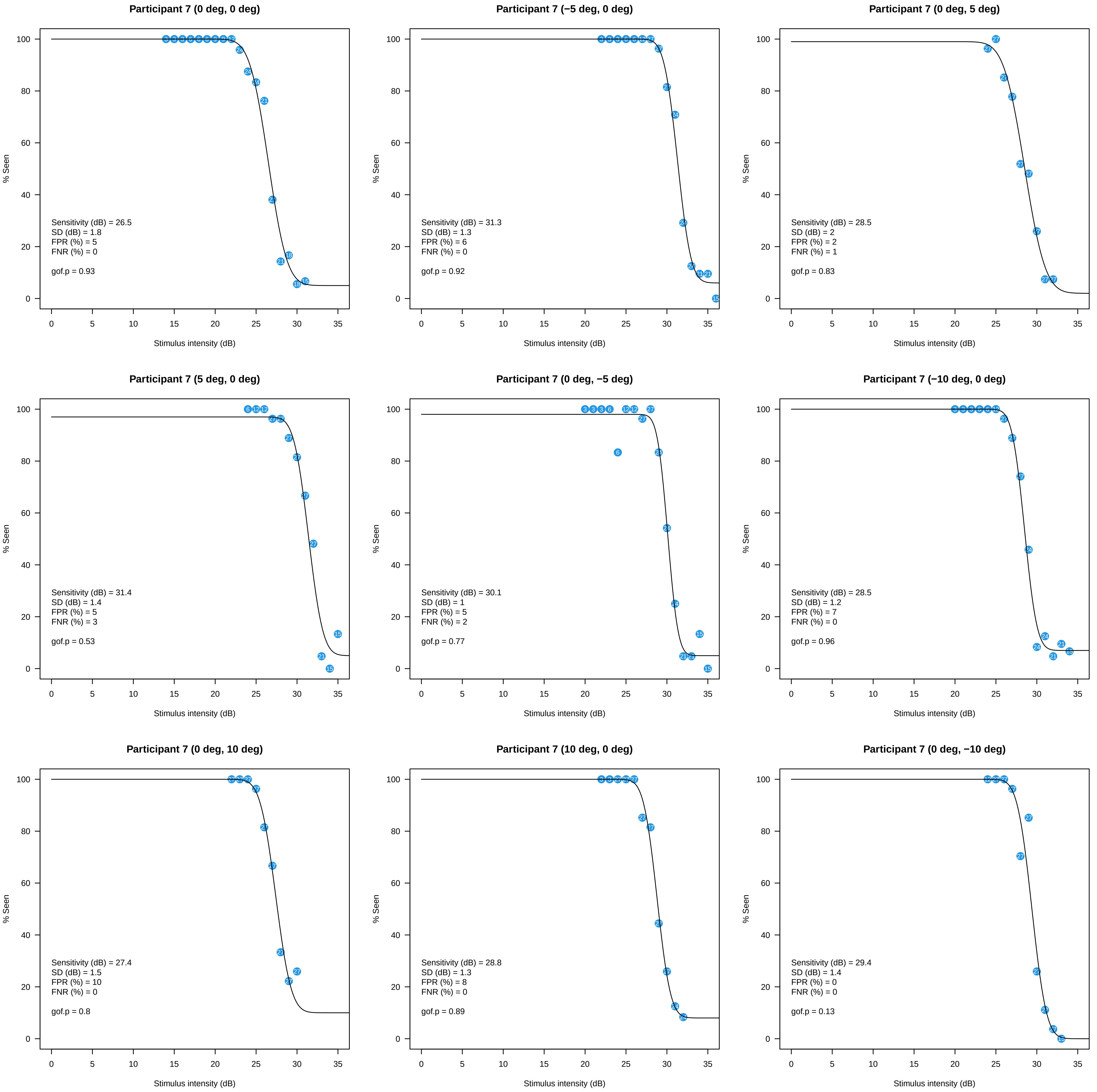

Participant 7 (0 deg, 0 deg)
Sensitivity (dB) = 26.5
SD (dB) = 1.8
FPR (%) = 5
FNR (%) = 0
gof.p = 0.93
Participant 7 (−5 deg, 0 deg)
Sensitivity (dB) = 31.3
SD (dB) = 1.3
FPR (%) = 6
FNR (%) = 0
gof.p = 0.92
Participant 7 (0 deg, 5 deg)
Sensitivity (dB) = 28.5
SD (dB) = 2
FPR (%) = 2
FNR (%) = 1
gof.p = 0.83
Participant 7 (5 deg, 0 deg)
Sensitivity (dB) = 31.4
SD (dB) = 1.4
FPR (%) = 5
FNR (%) = 3
gof.p = 0.53
Participant 7 (0 deg, −5 deg)
Sensitivity (dB) = 30.1
SD (dB) = 1
FPR (%) = 5
FNR (%) = 2
gof.p = 0.77
Participant 7 (−10 deg, 0 deg)
Sensitivity (dB) = 28.5
SD (dB) = 1.2
FPR (%) = 7
FNR (%) = 0
gof.p = 0.96
Participant 7 (0 deg, 10 deg)
Sensitivity (dB) = 27.4
SD (dB) = 1.5
FPR (%) = 10
FNR (%) = 0
gof.p = 0.8
Participant 7 (10 deg, 0 deg)
Sensitivity (dB) = 28.8
SD (dB) = 1.3
FPR (%) = 8
FNR (%) = 0
gof.p = 0.89
Participant 7 (0 deg, −10 deg)
Sensitivity (dB) = 29.4
SD (dB) = 1.4
FPR (%) = 0
FNR (%) = 0
gof.p = 0.13
% Seen
Stimulus intensity (dB)

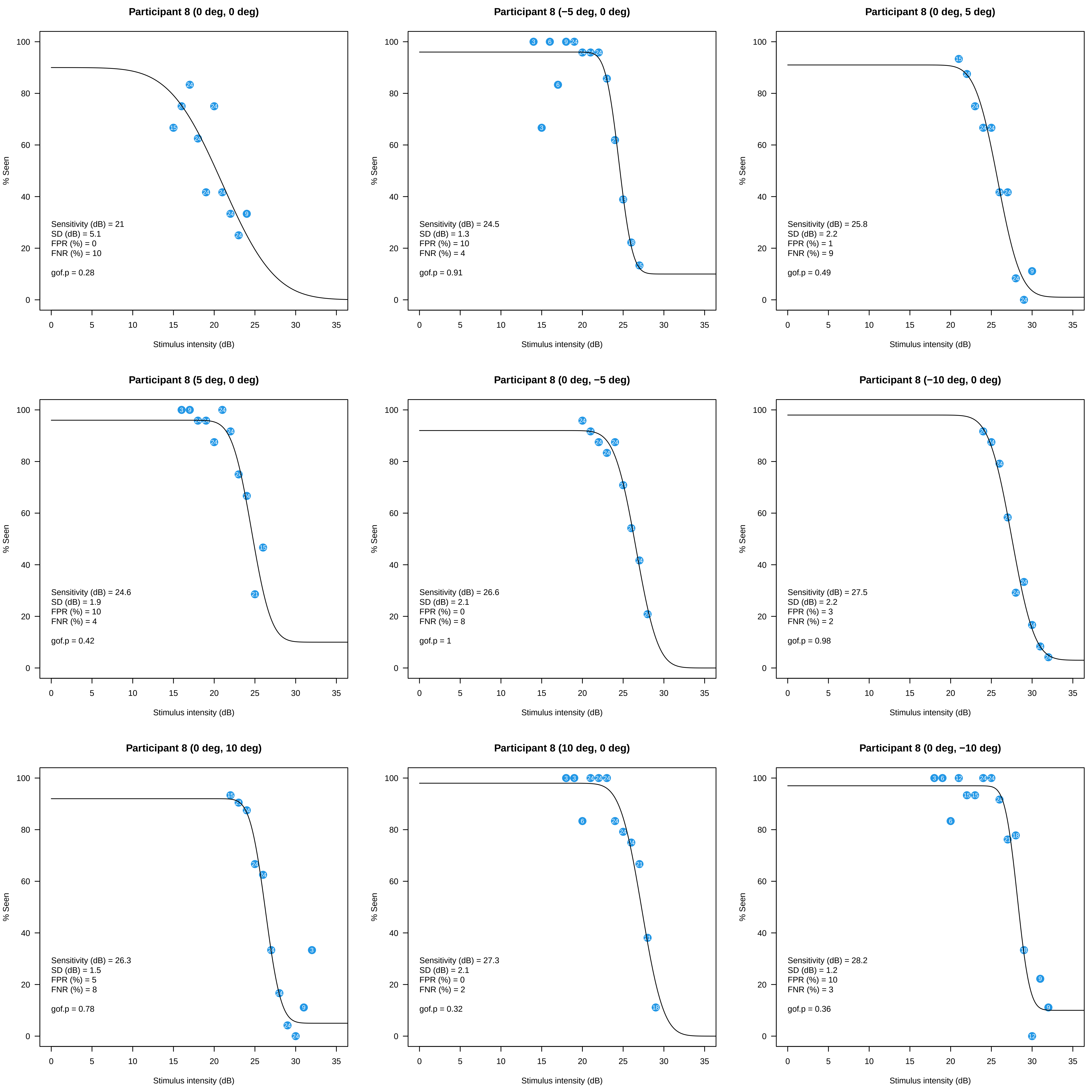
Participant 8 (0 deg, 0 deg)
Sensitivity (dB) = 21
SD (dB) = 5.1
FPR (%) = 0
FNR (%) = 10
gof.p = 0.28
Participant 8 (−5 deg, 0 deg)
Sensitivity (dB) = 24.5
SD (dB) = 1.3
FPR (%) = 10
FNR (%) = 4
gof.p = 0.91
Participant 8 (0 deg, 5 deg)
Sensitivity (dB) = 25.8
SD (dB) = 2.2
FPR (%) = 1
FNR (%) = 9
gof.p = 0.49
Participant 8 (5 deg, 0 deg)
Sensitivity (dB) = 24.6
SD (dB) = 1.9
FPR (%) = 10
FNR (%) = 4
gof.p = 0.42
Participant 8 (0 deg, −5 deg)
Sensitivity (dB) = 26.6
SD (dB) = 2.1
FPR (%) = 0
FNR (%) = 8
gof.p = 1
Participant 8 (−10 deg, 0 deg)
Sensitivity (dB) = 27.5
SD (dB) = 2.2
FPR (%) = 3
FNR (%) = 2
gof.p = 0.98
Participant 8 (0 deg, 10 deg)
Sensitivity (dB) = 26.3
SD (dB) = 1.5
FPR (%) = 5
FNR (%) = 8
gof.p = 0.78
Participant 8 (10 deg, 0 deg)
Sensitivity (dB) = 27.3
SD (dB) = 2.1
FPR (%) = 0
FNR (%) = 2
gof.p = 0.32
Participant 8 (0 deg, −10 deg)
Sensitivity (dB) = 28.2
SD (dB) = 1.2
FPR (%) = 10
FNR (%) = 3
gof.p = 0.36
% Seen
Stimulus intensity (dB)

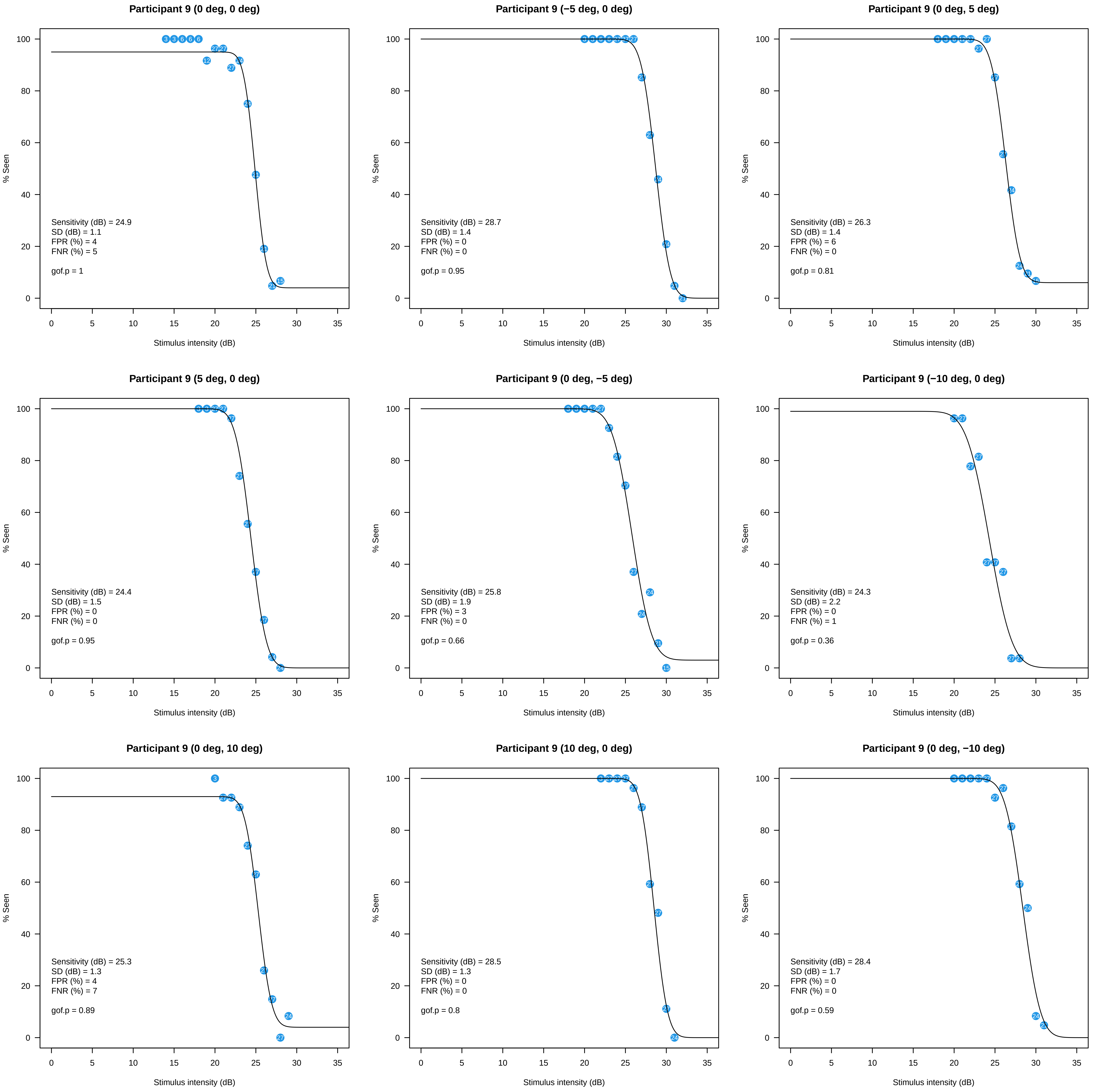
Participant 9 (0 deg, 0 deg)
Sensitivity (dB) = 24.9
SD (dB) = 1.1
FPR (%) = 4
FNR (%) = 5
gof.p = 1
Participant 9 (−5 deg, 0 deg)
Sensitivity (dB) = 28.7
SD (dB) = 1.4
FPR (%) = 0
FNR (%) = 0
gof.p = 0.95
Participant 9 (0 deg, 5 deg)
Sensitivity (dB) = 26.3
SD (dB) = 1.4
FPR (%) = 6
FNR (%) = 0
gof.p = 0.81
Participant 9 (5 deg, 0 deg)
Sensitivity (dB) = 24.4
SD (dB) = 1.5
FPR (%) = 0
FNR (%) = 0
gof.p = 0.95
Participant 9 (0 deg, −5 deg)
Sensitivity (dB) = 25.8
SD (dB) = 1.9
FPR (%) = 3
FNR (%) = 0
gof.p = 0.66
Participant 9 (−10 deg, 0 deg)
Sensitivity (dB) = 24.3
SD (dB) = 2.2
FPR (%) = 0
FNR (%) = 1
gof.p = 0.36
Participant 9 (0 deg, 10 deg)
Sensitivity (dB) = 25.3
SD (dB) = 1.3
FPR (%) = 4
FNR (%) = 7
gof.p = 0.89
Participant 9 (10 deg, 0 deg)
Sensitivity (dB) = 28.5
SD (dB) = 1.3
FPR (%) = 0
FNR (%) = 0
gof.p = 0.8
Participant 9 (0 deg, −10 deg)
Sensitivity (dB) = 28.4
SD (dB) = 1.7
FPR (%) = 0
FNR (%) = 0
gof.p = 0.59
% Seen
Stimulus intensity (dB)

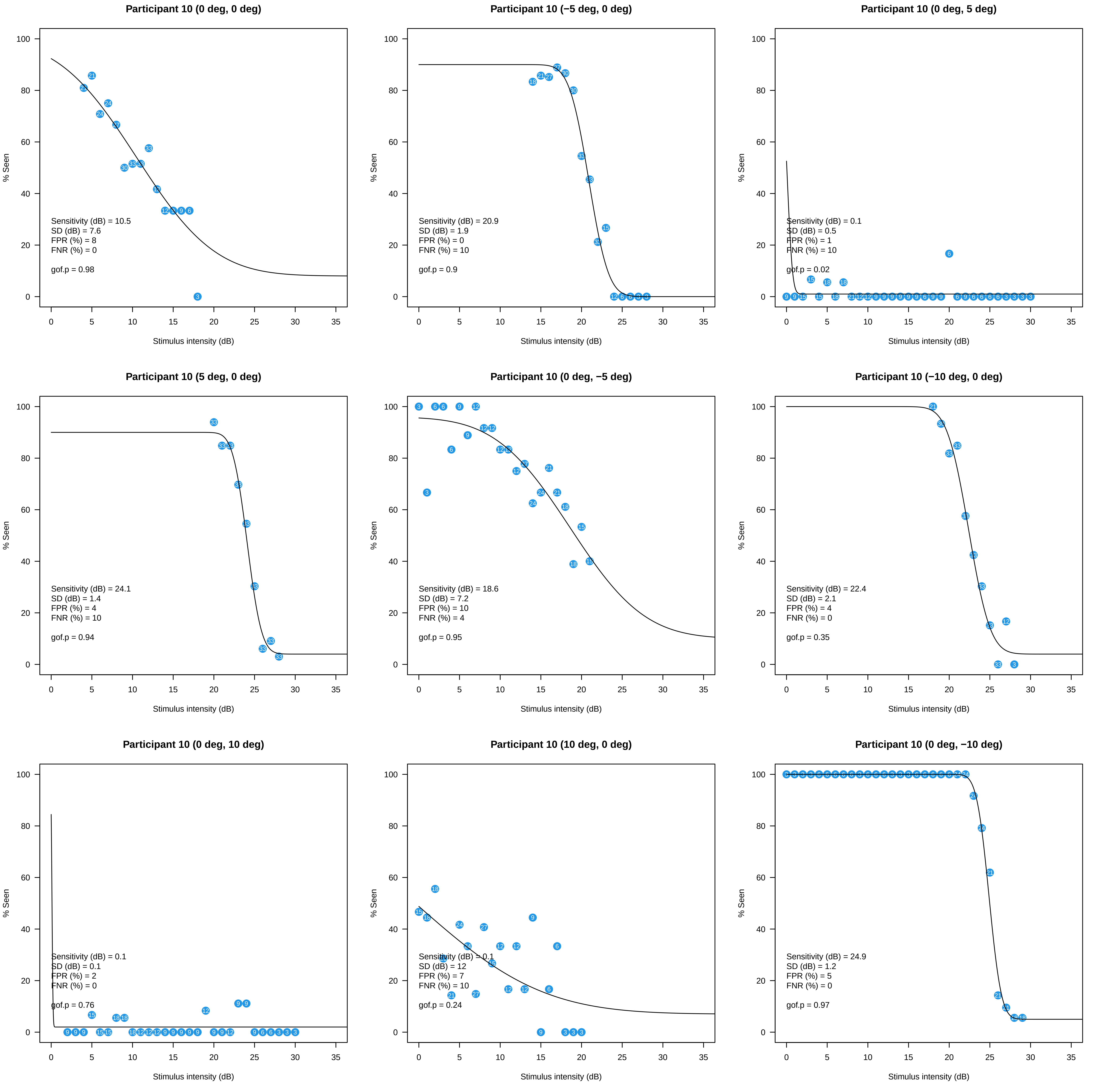
Participant 10 (0 deg, 0 deg)
Sensitivity (dB) = 10.5
SD (dB) = 7.6
FPR (%) = 8
FNR (%) = 0
gof.p = 0.98
Participant 10 (−5 deg, 0 deg)
Sensitivity (dB) = 20.9
SD (dB) = 1.9
FPR (%) = 0
FNR (%) = 10
gof.p = 0.9
Participant 10 (0 deg, 5 deg)
Sensitivity (dB) = 0.1
SD (dB) = 0.5
FPR (%) = 1
FNR (%) = 10
gof.p = 0.02
Participant 10 (5 deg, 0 deg)
Sensitivity (dB) = 24.1
SD (dB) = 1.4
FPR (%) = 4
FNR (%) = 10
gof.p = 0.94
Participant 10 (0 deg, −5 deg)
Sensitivity (dB) = 18.6
SD (dB) = 7.2
FPR (%) = 10
FNR (%) = 4
gof.p = 0.95
Participant 10 (−10 deg, 0 deg)
Sensitivity (dB) = 22.4
SD (dB) = 2.1
FPR (%) = 4
FNR (%) = 0
gof.p = 0.35
Participant 10 (0 deg, 10 deg)
Sensitivity (dB) = 0.1
SD (dB) = 0.1
FPR (%) = 2
FNR (%) = 0
gof.p = 0.76
Participant 10 (10 deg, 0 deg)
Sensitivity (dB) = 0.1
SD (dB) = 12
FPR (%) = 7
FNR (%) = 10
gof.p = 0.24
Participant 10 (0 deg, −10 deg)
Sensitivity (dB) = 24.9
SD (dB) = 1.2
FPR (%) = 5
FNR (%) = 0
gof.p = 0.97
% Seen
Stimulus intensity (dB)

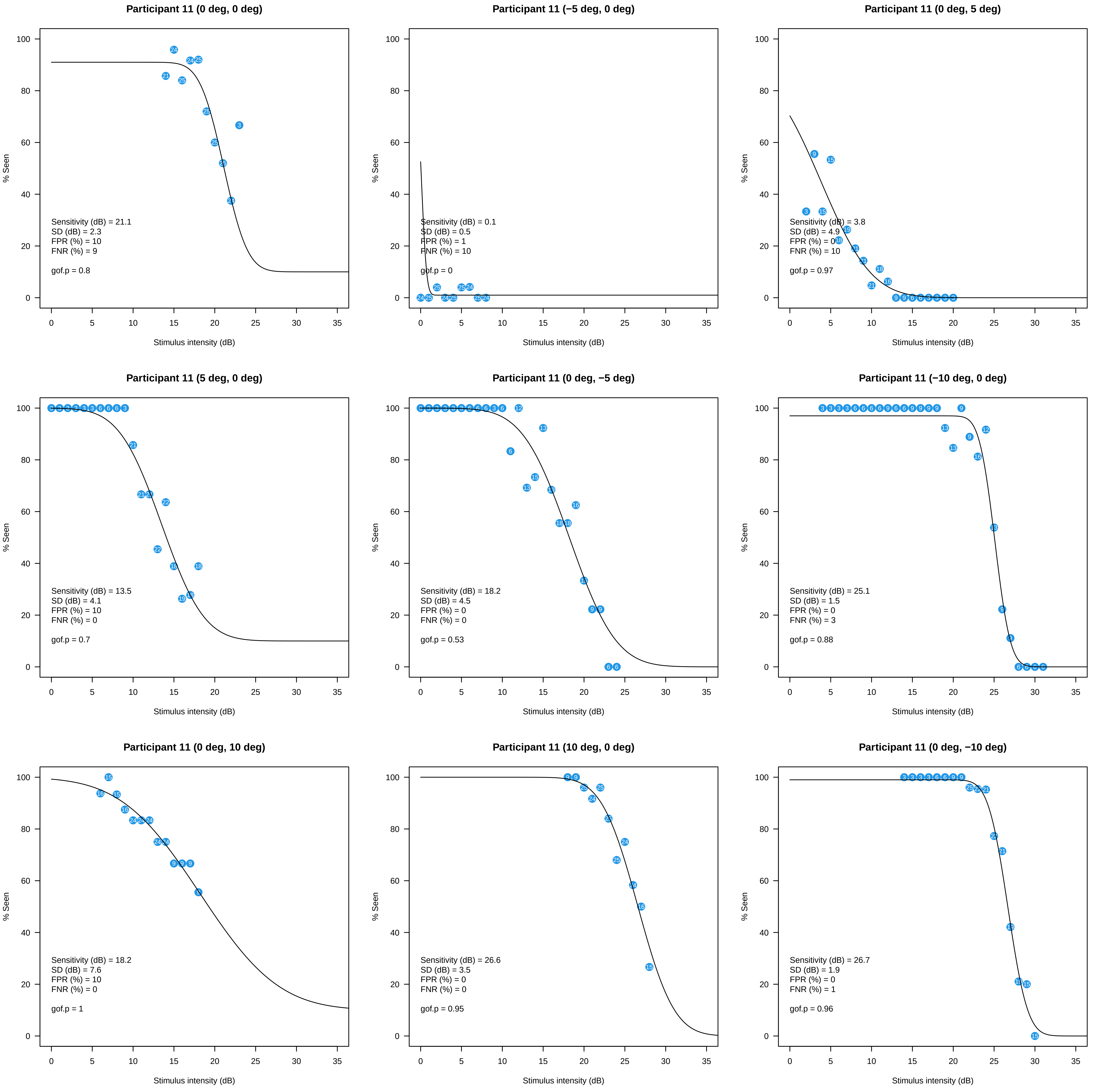
Participant 11 (0 deg, 0 deg)
Sensitivity (dB) = 21.1
SD (dB) = 2.3
FPR (%) = 10
FNR (%) = 9
gof.p = 0.8
Participant 11 (−5 deg, 0 deg)
Sensitivity (dB) = 0.1
SD (dB) = 0.5
FPR (%) = 1
FNR (%) = 10
gof.p = 0
Participant 11 (0 deg, 5 deg)
Sensitivity (dB) = 3.8
SD (dB) = 4.9
FPR (%) = 0
FNR (%) = 10
gof.p = 0.97
Participant 11 (5 deg, 0 deg)
Sensitivity (dB) = 13.5
SD (dB) = 4.1
FPR (%) = 10
FNR (%) = 0
gof.p = 0.7
Participant 11 (0 deg, −5 deg)
Sensitivity (dB) = 18.2
SD (dB) = 4.5
FPR (%) = 0
FNR (%) = 0
gof.p = 0.53
Participant 11 (−10 deg, 0 deg)
Sensitivity (dB) = 25.1
SD (dB) = 1.5
FPR (%) = 0
FNR (%) = 3
gof.p = 0.88
Participant 11 (0 deg, 10 deg)
Sensitivity (dB) = 18.2
SD (dB) = 7.6
FPR (%) = 10
FNR (%) = 0
gof.p = 1
Participant 11 (10 deg, 0 deg)
Sensitivity (dB) = 26.6
SD (dB) = 3.5
FPR (%) = 0
FNR (%) = 0
gof.p = 0.95
Participant 11 (0 deg, −10 deg)
Sensitivity (dB) = 26.7
SD (dB) = 1.9
FPR (%) = 0
FNR (%) = 1
gof.p = 0.96
% Seen
Stimulus intensity (dB)

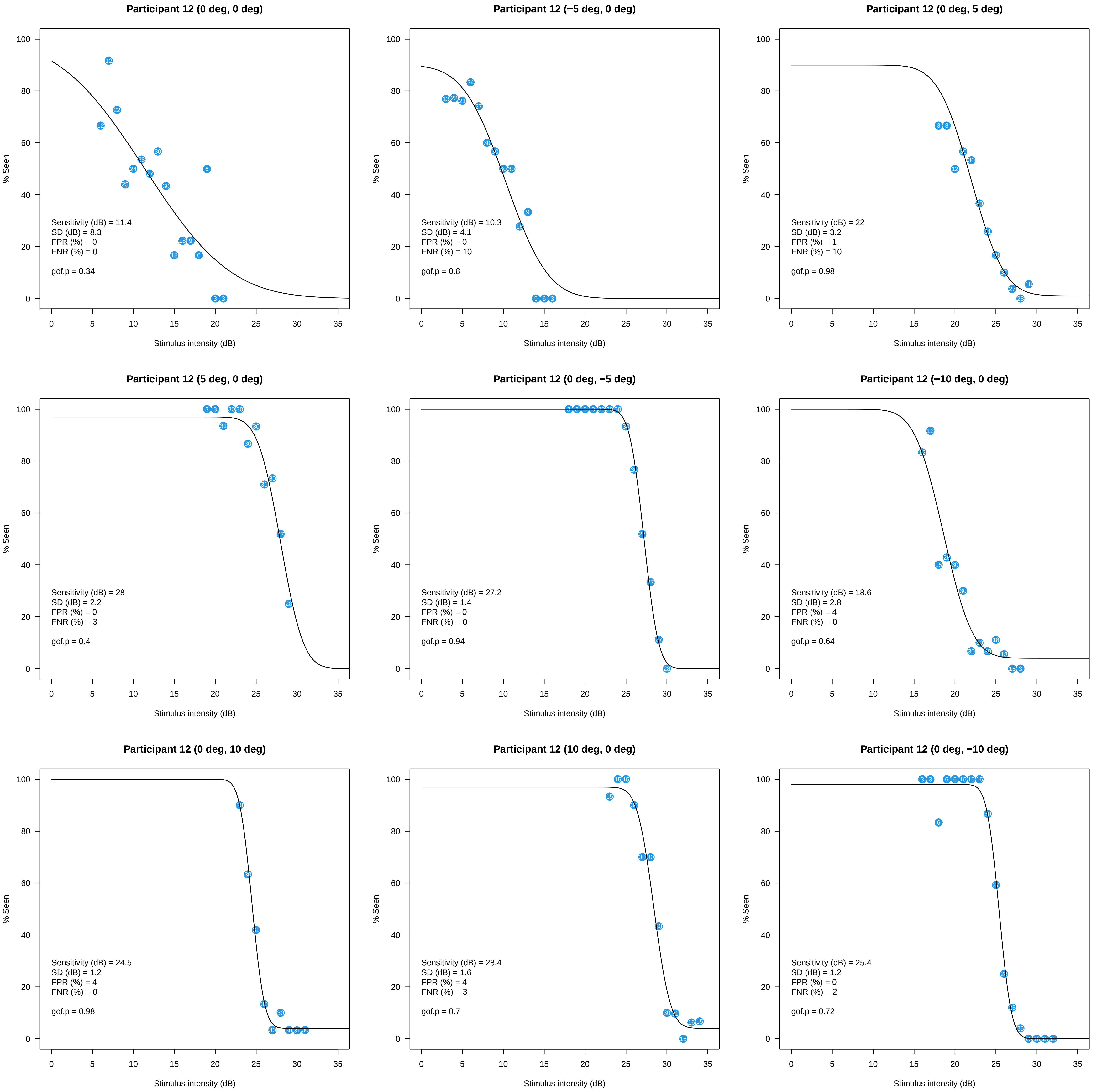

Participant 12 (0 deg, 0 deg)
Sensitivity (dB) = 11.4
SD (dB) = 8.3
FPR (%) = 0
FNR (%) = 0
gof.p = 0.34
Participant 12 (−5 deg, 0 deg)
Sensitivity (dB) = 10.3
SD (dB) = 4.1
FPR (%) = 0
FNR (%) = 10
gof.p = 0.8
Participant 12 (0 deg, 5 deg)
Sensitivity (dB) = 22
SD (dB) = 3.2
FPR (%) = 1
FNR (%) = 10
gof.p = 0.98
Participant 12 (5 deg, 0 deg)
Sensitivity (dB) = 28
SD (dB) = 2.2
FPR (%) = 0
FNR (%) = 3
gof.p = 0.4
Participant 12 (0 deg, −5 deg)
Sensitivity (dB) = 27.2
SD (dB) = 1.4
FPR (%) = 0
FNR (%) = 0
gof.p = 0.94
Participant 12 (−10 deg, 0 deg)
Sensitivity (dB) = 18.6
SD (dB) = 2.8
FPR (%) = 4
FNR (%) = 0
gof.p = 0.64
Participant 12 (0 deg, 10 deg)
Sensitivity (dB) = 24.5
SD (dB) = 1.2
FPR (%) = 4
FNR (%) = 0
gof.p = 0.98
Participant 12 (10 deg, 0 deg)
Sensitivity (dB) = 28.4
SD (dB) = 1.6
FPR (%) = 4
FNR (%) = 3
gof.p = 0.7
Participant 12 (0 deg, −10 deg)
Sensitivity (dB) = 25.4
SD (dB) = 1.2
FPR (%) = 0
FNR (%) = 2
gof.p = 0.72
% Seen
Stimulus intensity (dB)

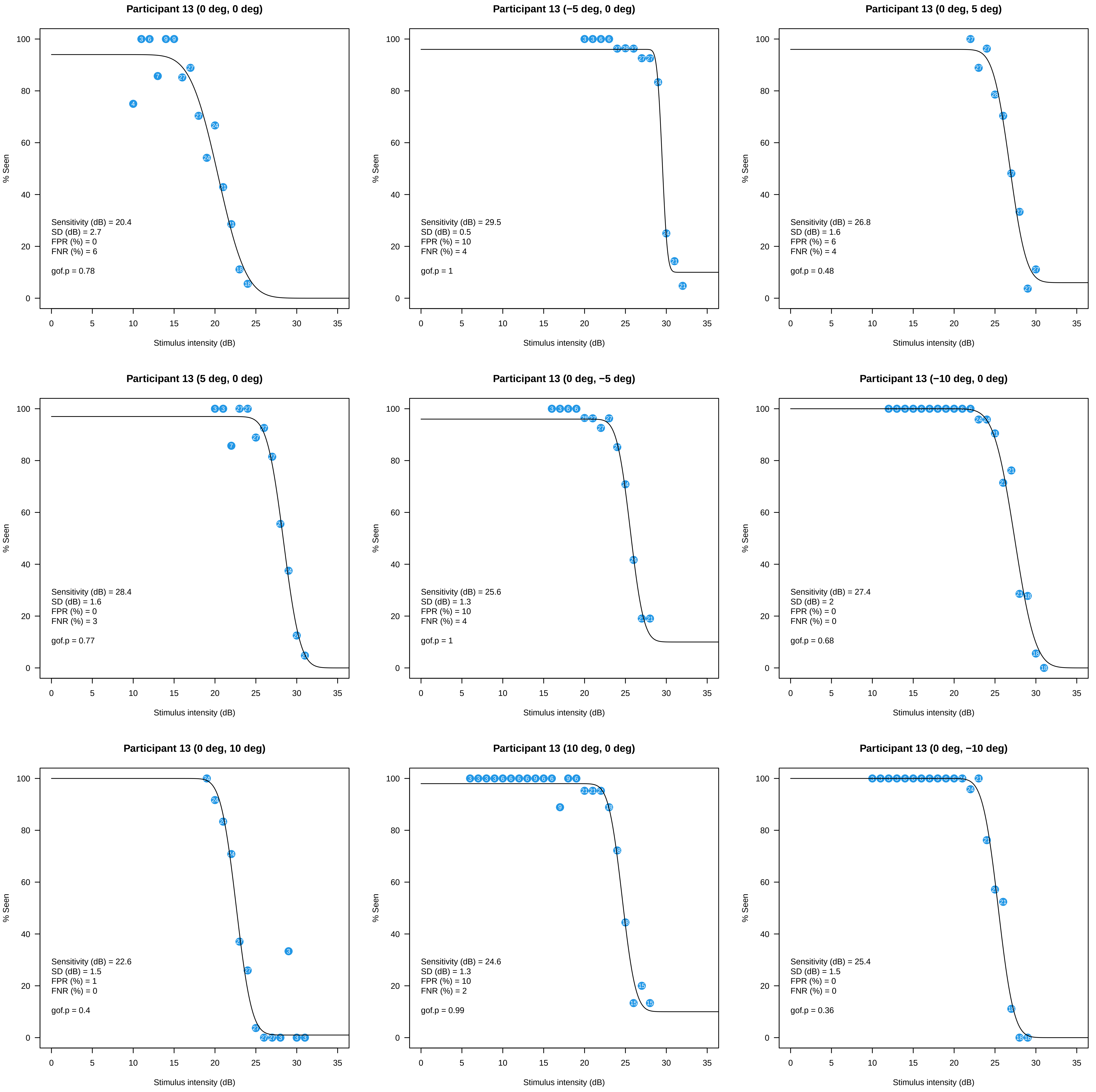
Participant 13 (0 deg, 0 deg)
Sensitivity (dB) = 20.4
SD (dB) = 2.7
FPR (%) = 0
FNR (%) = 6
gof.p = 0.78
Participant 13 (−5 deg, 0 deg)
Sensitivity (dB) = 29.5
SD (dB) = 0.5
FPR (%) = 10
FNR (%) = 4
gof.p = 1
Participant 13 (0 deg, 5 deg)
Sensitivity (dB) = 26.8
SD (dB) = 1.6
FPR (%) = 6
FNR (%) = 4
gof.p = 0.48
Participant 13 (5 deg, 0 deg)
Sensitivity (dB) = 28.4
SD (dB) = 1.6
FPR (%) = 0
FNR (%) = 3
gof.p = 0.77
Participant 13 (0 deg, −5 deg)
Sensitivity (dB) = 25.6
SD (dB) = 1.3
FPR (%) = 10
FNR (%) = 4
gof.p = 1
Participant 13 (−10 deg, 0 deg)
Sensitivity (dB) = 27.4
SD (dB) = 2
FPR (%) = 0
FNR (%) = 0
gof.p = 0.68
Participant 13 (0 deg, 10 deg)
Sensitivity (dB) = 22.6
SD (dB) = 1.5
FPR (%) = 1
FNR (%) = 0
gof.p = 0.4
Participant 13 (10 deg, 0 deg)
Sensitivity (dB) = 24.6
SD (dB) = 1.3
FPR (%) = 10
FNR (%) = 2
gof.p = 0.99
Participant 13 (0 deg, −10 deg)
Sensitivity (dB) = 25.4
SD (dB) = 1.5
FPR (%) = 0
FNR (%) = 0
gof.p = 0.36
% Seen
Stimulus intensity (dB)

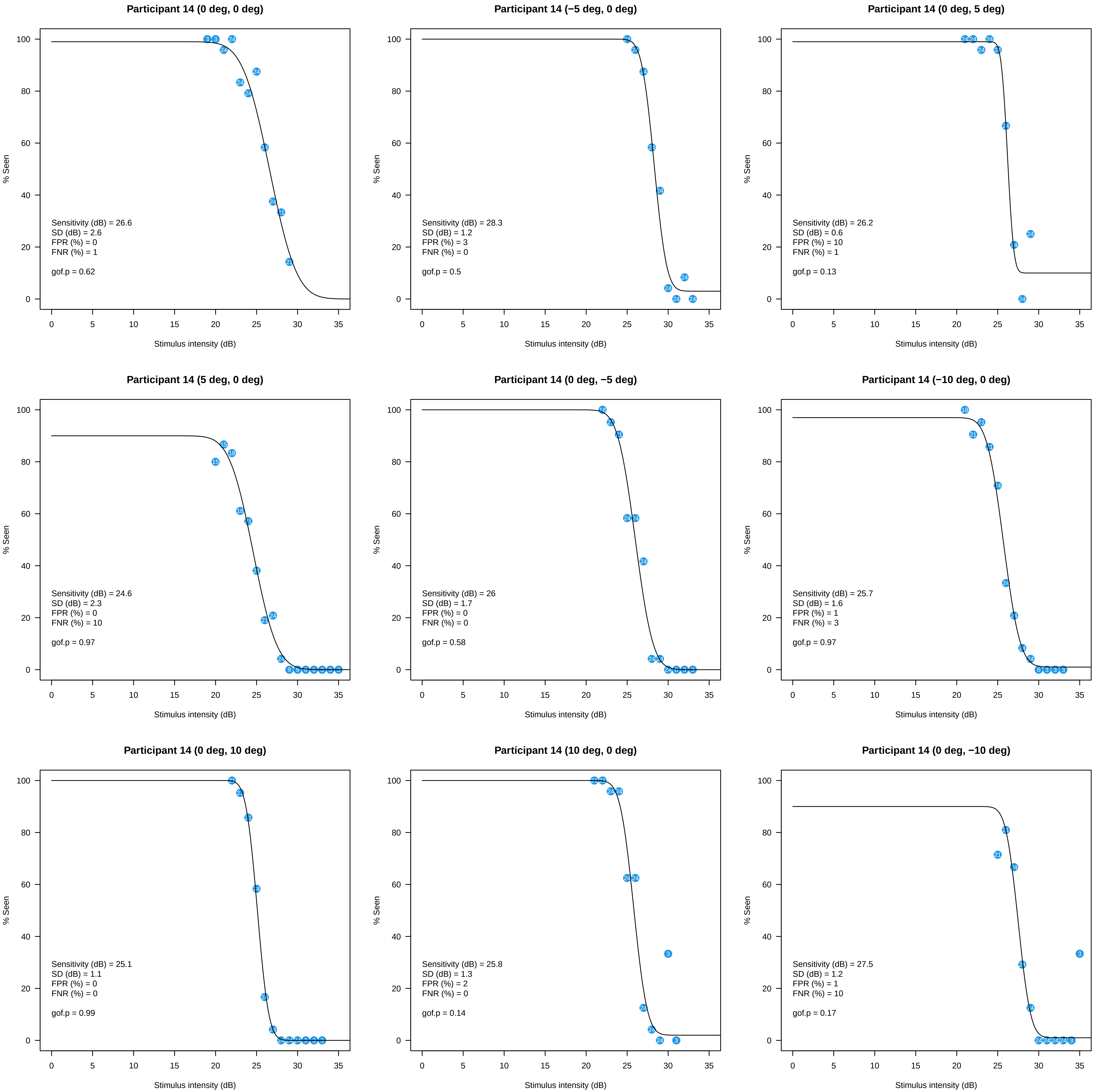
Participant 14 (0 deg, 0 deg)
Sensitivity (dB) = 26.6
SD (dB) = 2.6
FPR (%) = 0
FNR (%) = 1
gof.p = 0.62
Participant 14 (−5 deg, 0 deg)
Sensitivity (dB) = 28.3
SD (dB) = 1.2
FPR (%) = 3
FNR (%) = 0
gof.p = 0.5
Participant 14 (0 deg, 5 deg)
Sensitivity (dB) = 26.2
SD (dB) = 0.6
FPR (%) = 10
FNR (%) = 1
gof.p = 0.13
Participant 14 (5 deg, 0 deg)
Sensitivity (dB) = 24.6
SD (dB) = 2.3
FPR (%) = 0
FNR (%) = 10
gof.p = 0.97
Participant 14 (0 deg, −5 deg)
Sensitivity (dB) = 26
SD (dB) = 1.7
FPR (%) = 0
FNR (%) = 0
gof.p = 0.58
Participant 14 (−10 deg, 0 deg)
Sensitivity (dB) = 25.7
SD (dB) = 1.6
FPR (%) = 1
FNR (%) = 3
gof.p = 0.97
Participant 14 (0 deg, 10 deg)
Sensitivity (dB) = 25.1
SD (dB) = 1.1
FPR (%) = 0
FNR (%) = 0
gof.p = 0.99
Participant 14 (10 deg, 0 deg)
Sensitivity (dB) = 25.8
SD (dB) = 1.3
FPR (%) = 2
FNR (%) = 0
gof.p = 0.14
Participant 14 (0 deg, −10 deg)
Sensitivity (dB) = 27.5
SD (dB) = 1.2
FPR (%) = 1
FNR (%) = 10
gof.p = 0.17
% Seen
Stimulus intensity (dB)

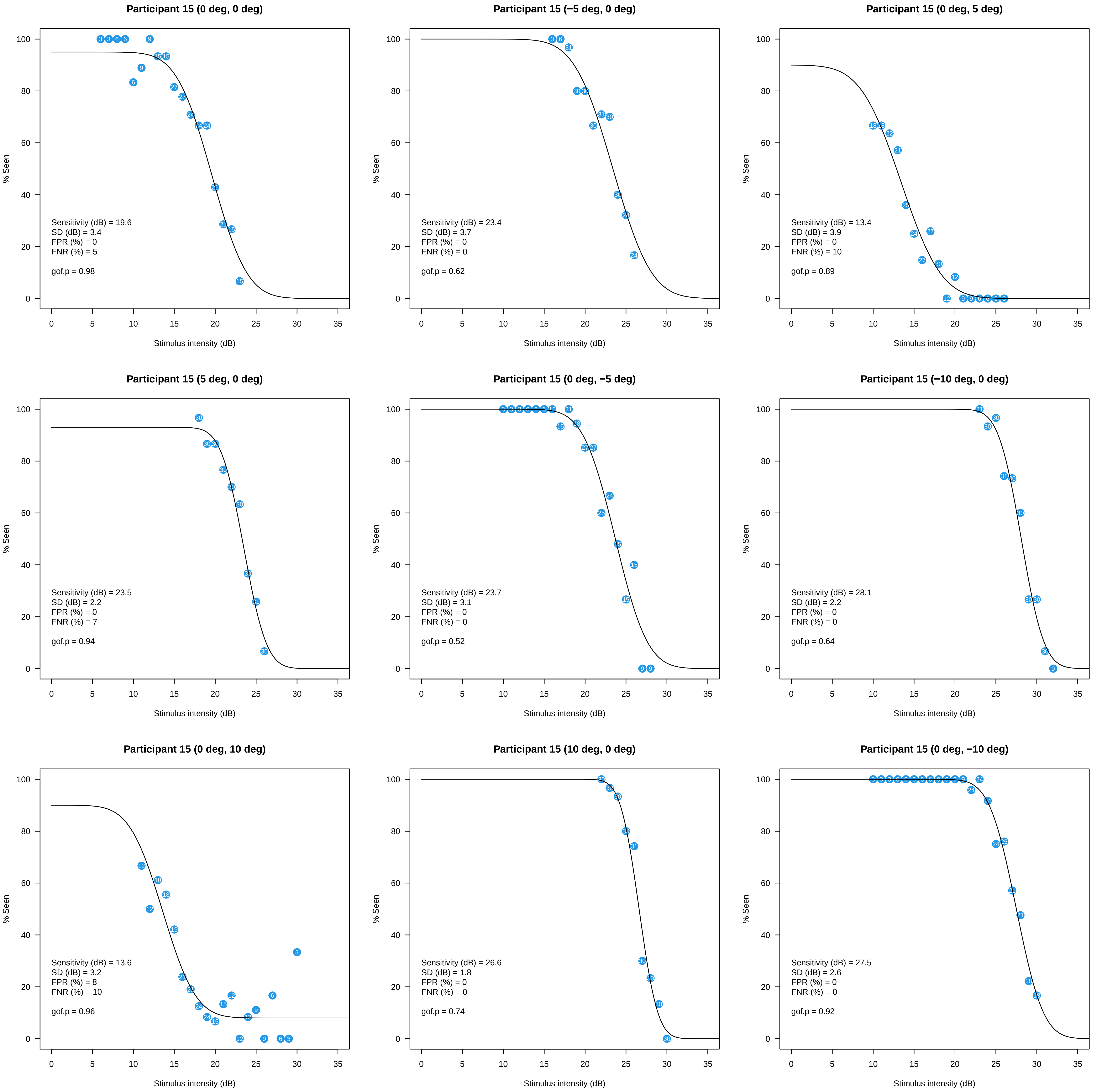
Participant 15 (0 deg, 0 deg)
Sensitivity (dB) = 19.6
SD (dB) = 3.4
FPR (%) = 0
FNR (%) = 5
gof.p = 0.98
Participant 15 (−5 deg, 0 deg)
Sensitivity (dB) = 23.4
SD (dB) = 3.7
FPR (%) = 0
FNR (%) = 0
gof.p = 0.62
Participant 15 (0 deg, 5 deg)
Sensitivity (dB) = 13.4
SD (dB) = 3.9
FPR (%) = 0
FNR (%) = 10
gof.p = 0.89
Participant 15 (5 deg, 0 deg)
Sensitivity (dB) = 23.5
SD (dB) = 2.2
FPR (%) = 0
FNR (%) = 7
gof.p = 0.94
Participant 15 (0 deg, −5 deg)
Sensitivity (dB) = 23.7
SD (dB) = 3.1
FPR (%) = 0
FNR (%) = 0
gof.p = 0.52
Participant 15 (−10 deg, 0 deg)
Sensitivity (dB) = 28.1
SD (dB) = 2.2
FPR (%) = 0
FNR (%) = 0
gof.p = 0.64
Participant 15 (0 deg, 10 deg)
Sensitivity (dB) = 13.6
SD (dB) = 3.2
FPR (%) = 8
FNR (%) = 10
gof.p = 0.96
Participant 15 (10 deg, 0 deg)
Sensitivity (dB) = 26.6
SD (dB) = 1.8
FPR (%) = 0
FNR (%) = 0
gof.p = 0.74
Participant 15 (0 deg, −10 deg)
Sensitivity (dB) = 27.5
SD (dB) = 2.6
FPR (%) = 0
FNR (%) = 0
gof.p = 0.92
% Seen
Stimulus intensity (dB)

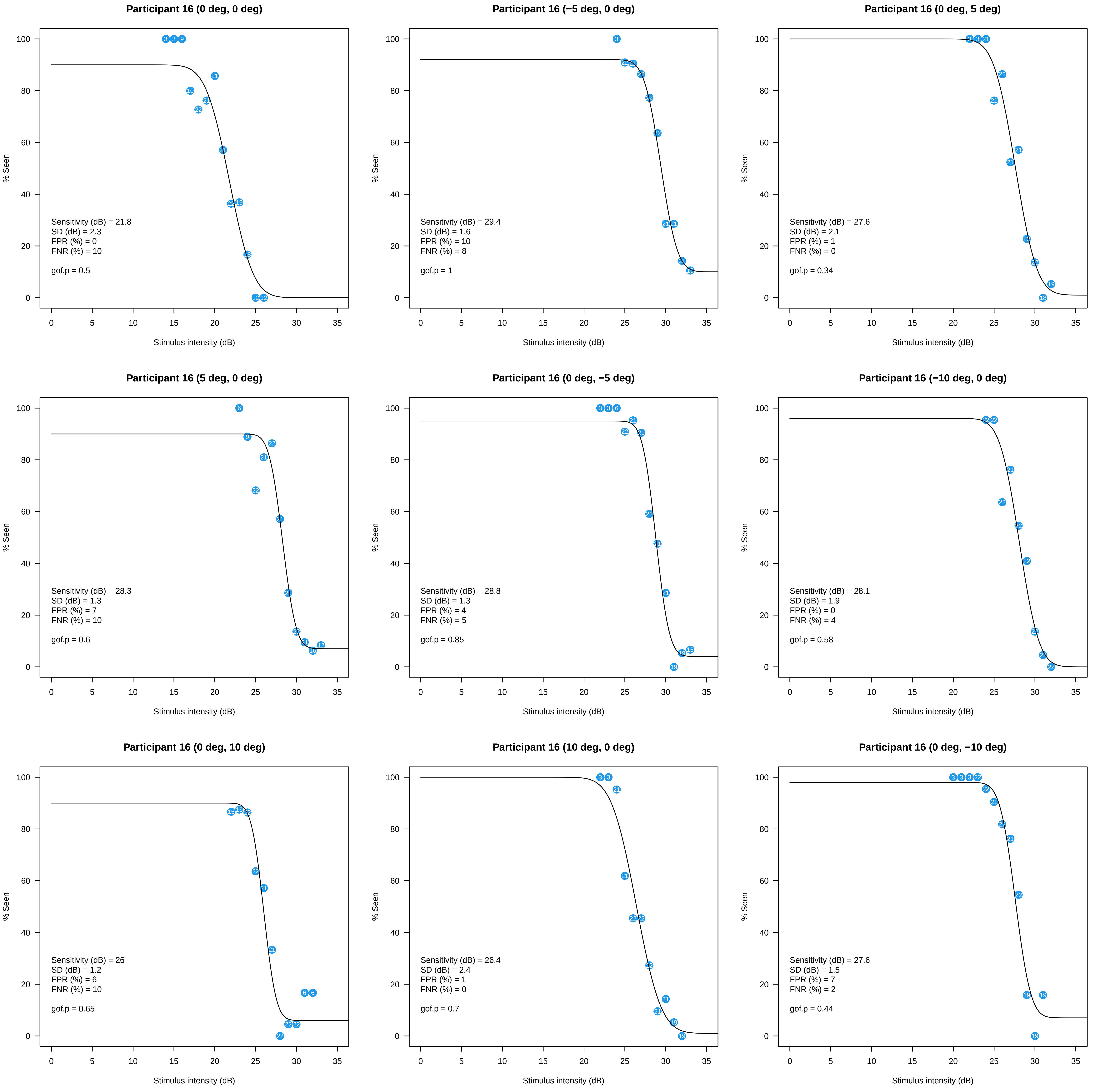
Participant 16 (0 deg, 0 deg)
Sensitivity (dB) = 21.8
SD (dB) = 2.3
FPR (%) = 0
FNR (%) = 10
gof.p = 0.5
Participant 16 (−5 deg, 0 deg)
Sensitivity (dB) = 29.4
SD (dB) = 1.6
FPR (%) = 10
FNR (%) = 8
gof.p = 1
Participant 16 (0 deg, 5 deg)
Sensitivity (dB) = 27.6
SD (dB) = 2.1
FPR (%) = 1
FNR (%) = 0
gof.p = 0.34
Participant 16 (5 deg, 0 deg)
Sensitivity (dB) = 28.3
SD (dB) = 1.3
FPR (%) = 7
FNR (%) = 10
gof.p = 0.6
Participant 16 (0 deg, −5 deg)
Sensitivity (dB) = 28.8
SD (dB) = 1.3
FPR (%) = 4
FNR (%) = 5
gof.p = 0.85
Participant 16 (−10 deg, 0 deg)
Sensitivity (dB) = 28.1
SD (dB) = 1.9
FPR (%) = 0
FNR (%) = 4
gof.p = 0.58
Participant 16 (0 deg, 10 deg)
Sensitivity (dB) = 26
SD (dB) = 1.2
FPR (%) = 6
FNR (%) = 10
gof.p = 0.65
Participant 16 (10 deg, 0 deg)
Sensitivity (dB) = 26.4
SD (dB) = 2.4
FPR (%) = 1
FNR (%) = 0
gof.p = 0.7
Participant 16 (0 deg, −10 deg)
Sensitivity (dB) = 27.6
SD (dB) = 1.5
FPR (%) = 7
FNR (%) = 2
gof.p = 0.44
% Seen
Stimulus intensity (dB)

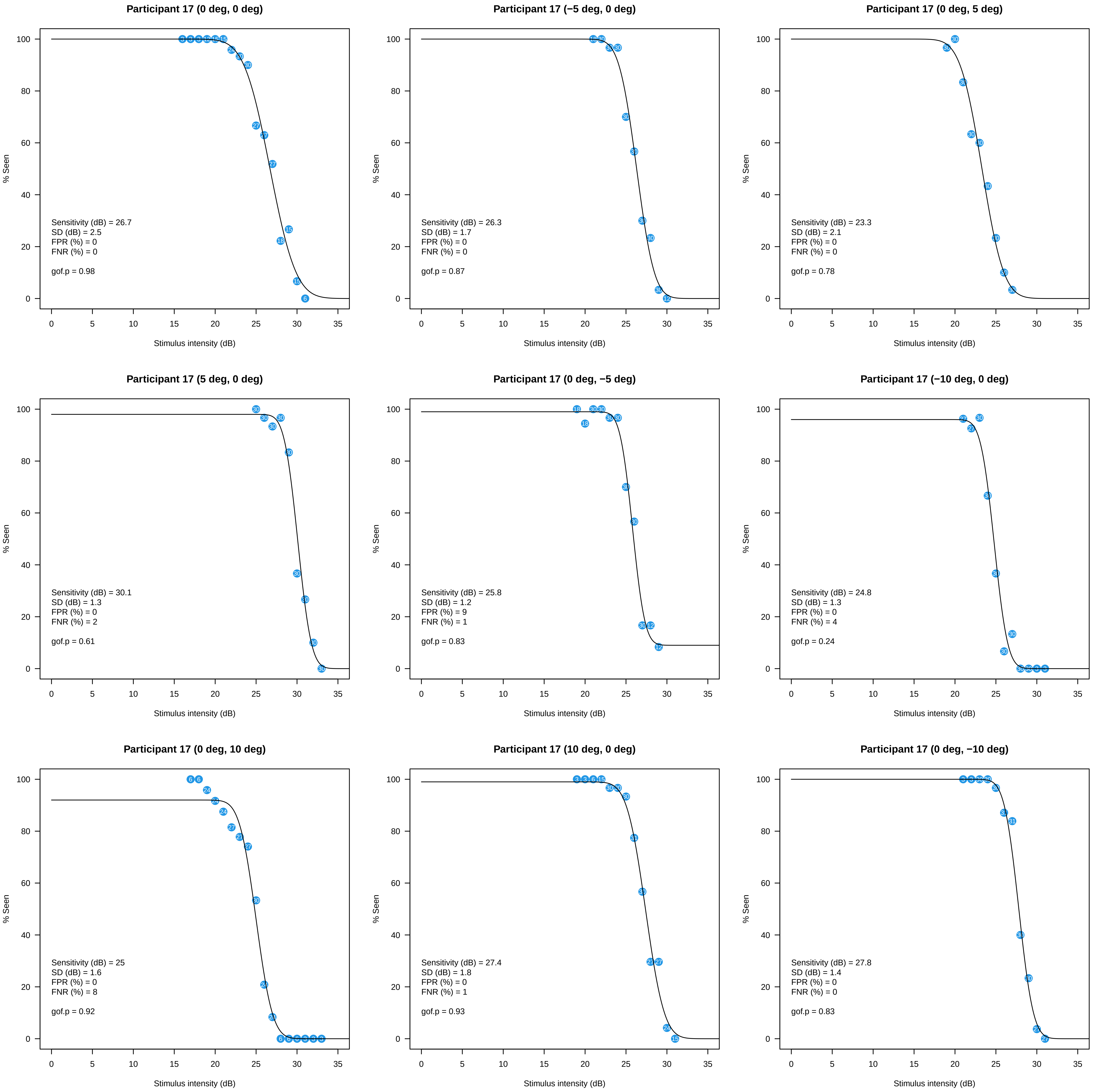

Participant 17 (0 deg, 0 deg)
Sensitivity (dB) = 26.7
SD (dB) = 2.5
FPR (%) = 0
FNR (%) = 0
gof.p = 0.98
Participant 17 (−5 deg, 0 deg)
Sensitivity (dB) = 26.3
SD (dB) = 1.7
FPR (%) = 0
FNR (%) = 0
gof.p = 0.87
Participant 17 (0 deg, 5 deg)
Sensitivity (dB) = 23.3
SD (dB) = 2.1
FPR (%) = 0
FNR (%) = 0
gof.p = 0.78
Participant 17 (5 deg, 0 deg)
Sensitivity (dB) = 30.1
SD (dB) = 1.3
FPR (%) = 0
FNR (%) = 2
gof.p = 0.61
Participant 17 (0 deg, −5 deg)
Sensitivity (dB) = 25.8
SD (dB) = 1.2
FPR (%) = 9
FNR (%) = 1
gof.p = 0.83
Participant 17 (−10 deg, 0 deg)
Sensitivity (dB) = 24.8
SD (dB) = 1.3
FPR (%) = 0
FNR (%) = 4
gof.p = 0.24
Participant 17 (0 deg, 10 deg)
Sensitivity (dB) = 25
SD (dB) = 1.6
FPR (%) = 0
FNR (%) = 8
gof.p = 0.92
Participant 17 (10 deg, 0 deg)
Sensitivity (dB) = 27.4
SD (dB) = 1.8
FPR (%) = 0
FNR (%) = 1
gof.p = 0.93
Participant 17 (0 deg, −10 deg)
Sensitivity (dB) = 27.8
SD (dB) = 1.4
FPR (%) = 0
FNR (%) = 0
gof.p = 0.83
% Seen
Stimulus intensity (dB)

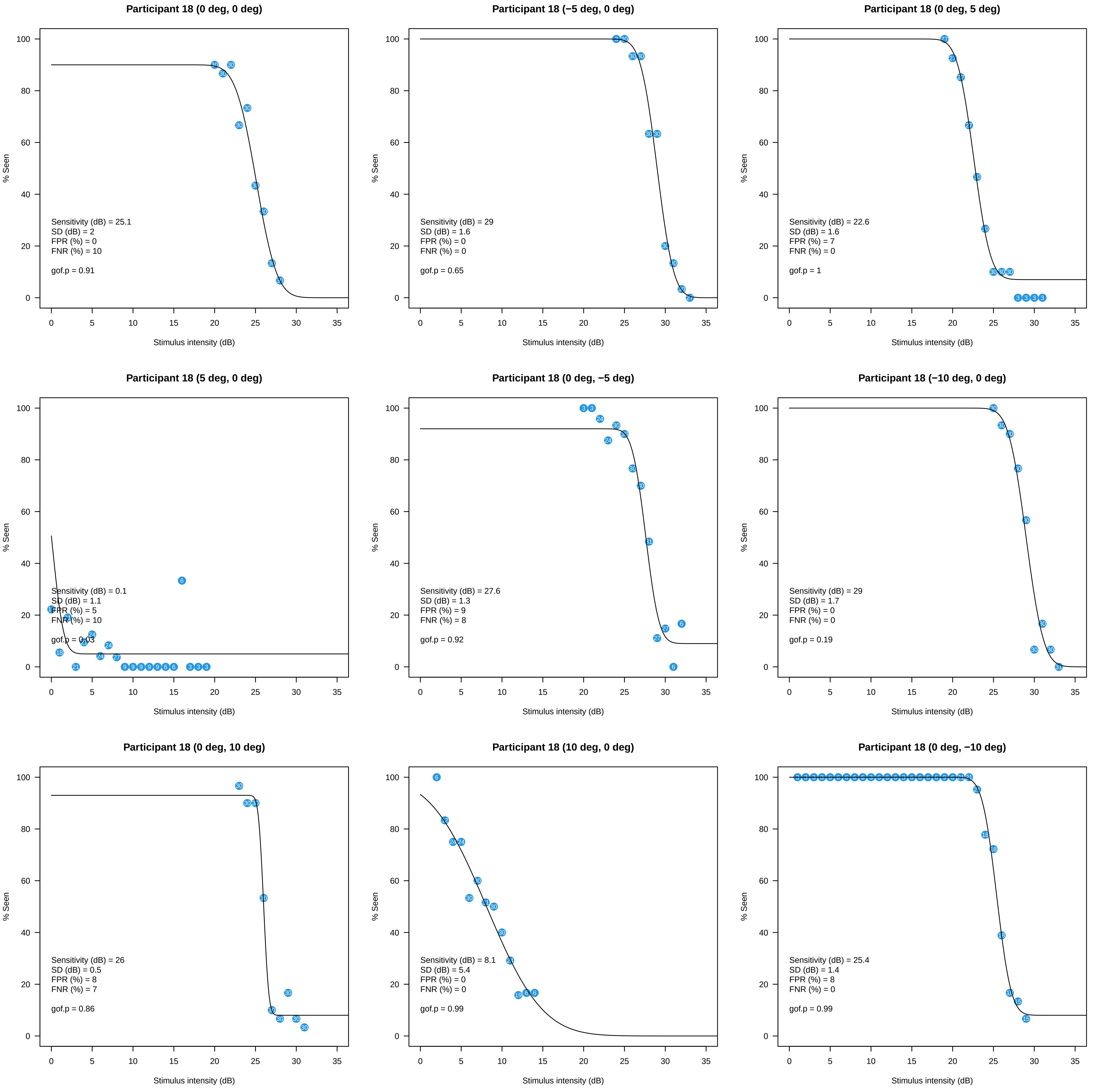
Participant 18 (0 deg, 0 deg)
Sensitivity (dB) = 25.1
SD (dB) = 2
FPR (%) = 0
FNR (%) = 10
gof.p = 0.91
Participant 18 (−5 deg, 0 deg)
Sensitivity (dB) = 29
SD (dB) = 1.6
FPR (%) = 0
FNR (%) = 0
gof.p = 0.65
Participant 18 (0 deg, 5 deg)
Sensitivity (dB) = 22.6
SD (dB) = 1.6
FPR (%) = 7
FNR (%) = 0
gof.p = 1
Participant 18 (5 deg, 0 deg)
Sensitivity (dB) = 0.1
SD (dB) = 1.1
FPR (%) = 5
FNR (%) = 10
gof.p = 0.03
Participant 18 (0 deg, −5 deg)
Sensitivity (dB) = 27.6
SD (dB) = 1.3
FPR (%) = 9
FNR (%) = 8
gof.p = 0.92
Participant 18 (−10 deg, 0 deg)
Sensitivity (dB) = 29
SD (dB) = 1.7
FPR (%) = 0
FNR (%) = 0
gof.p = 0.19
Participant 18 (0 deg, 10 deg)
Sensitivity (dB) = 26
SD (dB) = 0.5
FPR (%) = 8
FNR (%) = 7
gof.p = 0.86
Participant 18 (10 deg, 0 deg)
Sensitivity (dB) = 8.1
SD (dB) = 5.4
FPR (%) = 0
FNR (%) = 0
gof.p = 0.99
Participant 18 (0 deg, −10 deg)
Sensitivity (dB) = 25.4
SD (dB) = 1.4
FPR (%) = 8
FNR (%) = 0
gof.p = 0.99
% Seen
Stimulus intensity (dB)

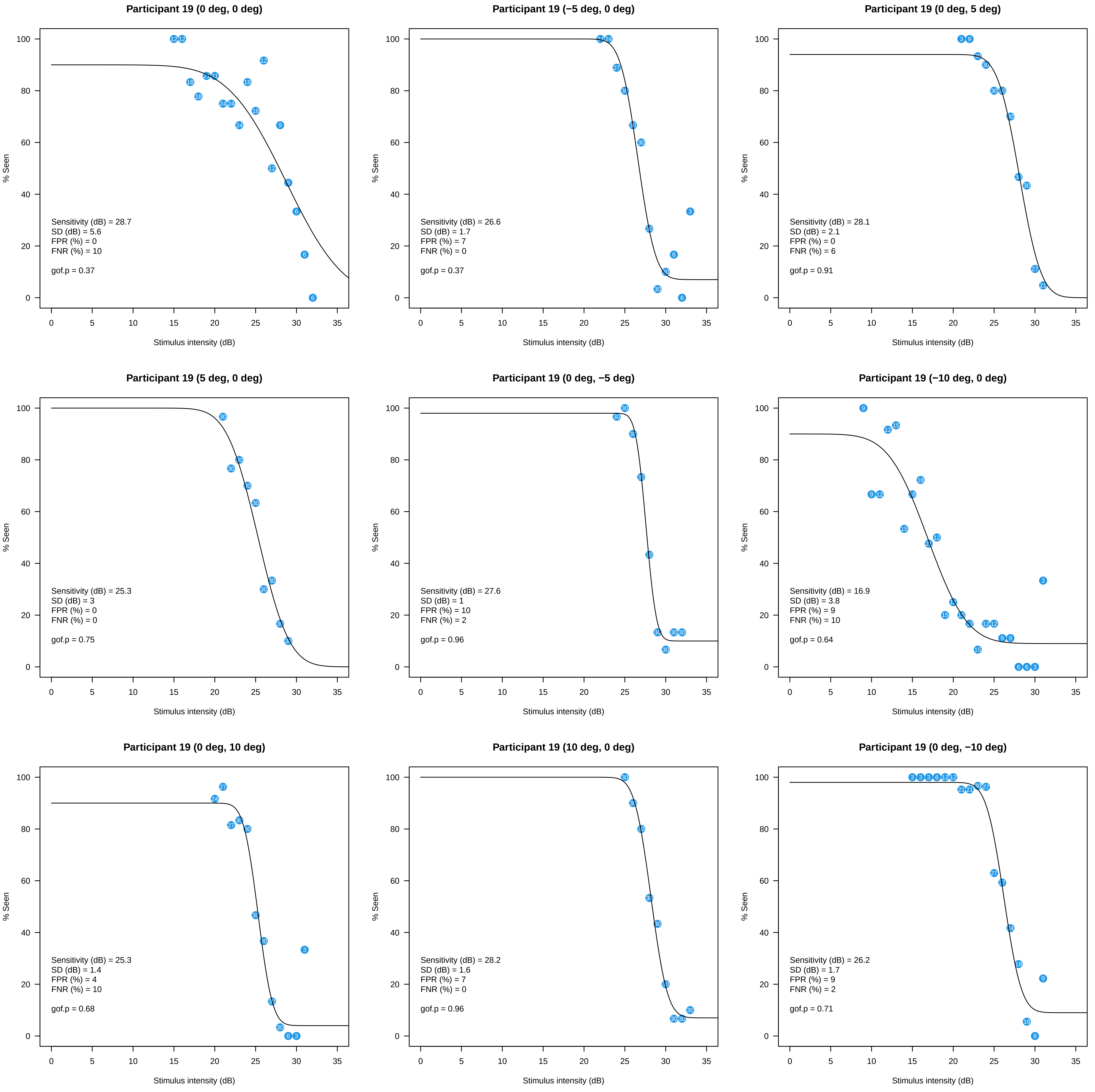

Participant 19 (0 deg, 0 deg)
Sensitivity (dB) = 28.7
SD (dB) = 5.6
FPR (%) = 0
FNR (%) = 10
gof.p = 0.37
Participant 19 (−5 deg, 0 deg)
Sensitivity (dB) = 26.6
SD (dB) = 1.7
FPR (%) = 7
FNR (%) = 0
gof.p = 0.37
Participant 19 (0 deg, 5 deg)
Sensitivity (dB) = 28.1
SD (dB) = 2.1
FPR (%) = 0
FNR (%) = 6
gof.p = 0.91
Participant 19 (5 deg, 0 deg)
Sensitivity (dB) = 25.3
SD (dB) = 3
FPR (%) = 0
FNR (%) = 0
gof.p = 0.75
Participant 19 (0 deg, −5 deg)
Sensitivity (dB) = 27.6
SD (dB) = 1
FPR (%) = 10
FNR (%) = 2
gof.p = 0.96
Participant 19 (−10 deg, 0 deg)
Sensitivity (dB) = 16.9
SD (dB) = 3.8
FPR (%) = 9
FNR (%) = 10
gof.p = 0.64
Participant 19 (0 deg, 10 deg)
Sensitivity (dB) = 25.3
SD (dB) = 1.4
FPR (%) = 4
FNR (%) = 10
gof.p = 0.68
Participant 19 (10 deg, 0 deg)
Sensitivity (dB) = 28.2
SD (dB) = 1.6
FPR (%) = 7
FNR (%) = 0
gof.p = 0.96
Participant 19 (0 deg, −10 deg)
Sensitivity (dB) = 26.2
SD (dB) = 1.7
FPR (%) = 9
FNR (%) = 2
gof.p = 0.71
% Seen
Stimulus intensity (dB)

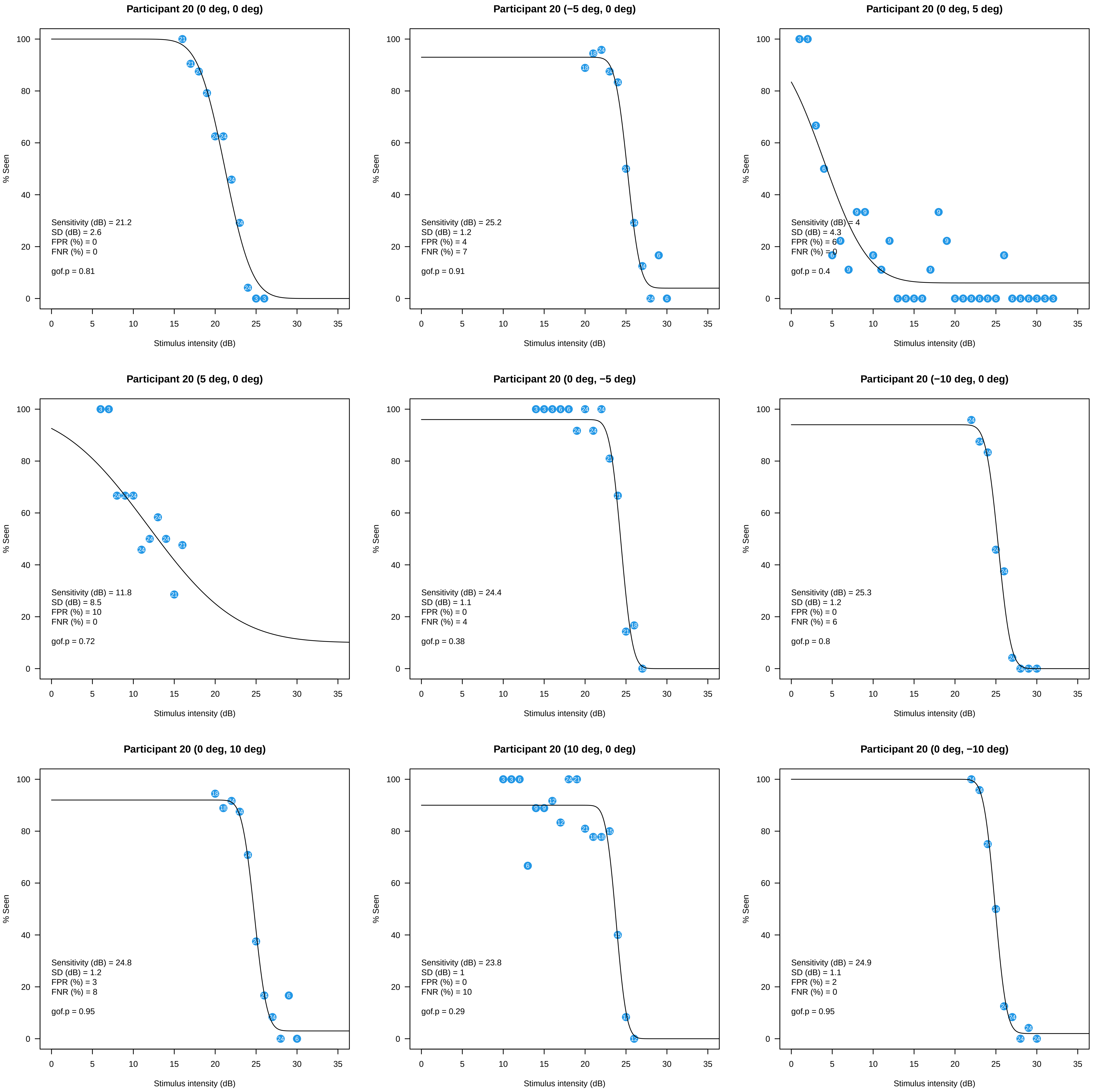
Participant 20 (0 deg, 0 deg)
Sensitivity (dB) = 21.2
SD (dB) = 2.6
FPR (%) = 0
FNR (%) = 0
gof.p = 0.81
Participant 20 (−5 deg, 0 deg)
Sensitivity (dB) = 25.2
SD (dB) = 1.2
FPR (%) = 4
FNR (%) = 7
gof.p = 0.91
Participant 20 (0 deg, 5 deg)
Sensitivity (dB) = 4
SD (dB) = 4.3
FPR (%) = 6
FNR (%) = 0
gof.p = 0.4
Participant 20 (5 deg, 0 deg)
Sensitivity (dB) = 11.8
SD (dB) = 8.5
FPR (%) = 10
FNR (%) = 0
gof.p = 0.72
Participant 20 (0 deg, −5 deg)
Sensitivity (dB) = 24.4
SD (dB) = 1.1
FPR (%) = 0
FNR (%) = 4
gof.p = 0.38
Participant 20 (−10 deg, 0 deg)
Sensitivity (dB) = 25.3
SD (dB) = 1.2
FPR (%) = 0
FNR (%) = 6
gof.p = 0.8
Participant 20 (0 deg, 10 deg)
Sensitivity (dB) = 24.8
SD (dB) = 1.2
FPR (%) = 3
FNR (%) = 8
gof.p = 0.95
Participant 20 (10 deg, 0 deg)
Sensitivity (dB) = 23.8
SD (dB) = 1
FPR (%) = 0
FNR (%) = 10
gof.p = 0.29
Participant 20 (0 deg, −10 deg)
Sensitivity (dB) = 24.9
SD (dB) = 1.1
FPR (%) = 2
FNR (%) = 0
gof.p = 0.95
% Seen
Stimulus intensity (dB)
